# Design, In Silico, and Experimental Evaluation of Novel Naproxen–Azetidinone Hybrids as Selective COX-2 Inhibitors

**DOI:** 10.3390/molecules30224358

**Published:** 2025-11-11

**Authors:** Ayad Kareem Khan, Noor Riyadh Mahmood, Mohammed Abdulaali Sahib

**Affiliations:** 1 Department of Pharmaceutical Chemistry, College of Pharmacy, Mustansiriyah University, Baghdad 10052, Iraq; noor92@uomustansiriyah.edu.iq; 2 Department of Pharmaceutical Chemistry, College of Pharmacy, University of Kerbala, Karbala 56001, Iraq; mohammed.a.sahib@uokerbala.edu.iq

**Keywords:** molecular dynamics simulation, azetidinone hybrids, anti-inflammatory activity, molecular docking, MM/GBSA, COX-2 inhibition, DFT analysis

## Abstract

The therapeutic use of non-steroidal anti-inflammatory drugs (NSAIDs) is limited by gastrointestinal and renal adverse effects caused by non-selective COX-1 and COX-2 inhibition. To address this issue, a new series of naproxen–azetidinone hybrids was rationally designed and synthesized to enhance COX-2 selectivity and reduce off-target toxicity. The synthesis involved esterification, hydrazide formation, Schiff base condensation, and intramolecular cyclization with chloroacetyl chloride. Structural characterization was achieved through FT-IR, ^1^H NMR, and ^13^C NMR analyses. In silico ADMET profiling confirmed compliance with Lipinski’s rule and predicted favorable gastrointestinal absorption. Molecular docking revealed high COX-2 binding affinities (−11.93 to −9.72 kcal/mol), while MM/GBSA analysis identified compound **N4_c_** (ΔG = −62.27 kcal/mol) as the most stable complex, surpassing meloxicam and naproxen. DFT (B3LYP/6-31G(d,p)) frontier molecular orbital analysis indicated a narrow HOMO–LUMO gap (ΔE = 2.97 eV) for **N4_c_**, suggesting high electronic reactivity and strong enzyme interaction. Molecular dynamics simulations confirmed complex stability. In vivo anti-inflammatory testing using an egg-white-induced rat paw edema model showed that **N4_d_**, **N4_e_**, and **N4_f_** achieved higher inhibition (19.22%, 16.98%, and 16.98%) than naproxen (4.3%). These results highlight 2-azetidinone–naproxen hybrids as promising selective COX-2 inhibitors with enhanced pharmacokinetic and electronic properties.

## 1. Introduction

Inflammation is a natural biological response that occurs in vascular tissues when the body is exposed to infections, cell damage, or chemical irritants. It takes care of the body by eliminating harmful elements and repairing and regenerating damaged tissue. There are generally two categories of inflammation: acute inflammation and chronic inflammation, which are distinguished by their duration and cause [1].

Acute inflammation refers to the response of the natural immune system of the body in response to injury or infection. It may occur rapidly and can be extremely severe, as seen in illnesses such as sinusitis, tonsillitis, and acute bronchitis. Chronic inflammation, on the other hand, is often associated with diseases such as tuberculosis and atherosclerosis. It can occur due to autoimmune responses, prolonged exposure to irritants, a chronic infection, or an inability to eliminate the underlying causes [2,3].

Patients often use nonsteroidal anti-inflammatory medicines (NSAIDs) to alleviate mild to moderate pain and other inflammatory conditions, such as arthritis and rheumatism. Nonsteroidal anti-inflammatory drugs (NSAIDs) essentially block cyclooxygenase enzymes (COX-1 and COX-2), which promote the production of thromboxanes and prostaglandins associated with inflammation, fever, and discomfort. Prior research has suggested that selective COX-2 inhibition may provide analgesic and anti-inflammatory benefits without the adverse effects associated with COX-1 inhibition [4]. The kind and intensity of adverse effects vary with medicine, often including hemorrhage, gastrointestinal ulcers, and an elevated risk of cardiovascular and renal problems [5].

Naproxen, an extensively used derivative of propionic acid, works by blocking the COX enzymes that produce prostaglandins, which effectively lowers pain, swelling, and arthritic symptoms. However, naproxen’s free carboxylic acid group is primarily responsible for the gastrointestinal adverse effects linked to its use. As a result, hiding this acidic component has been suggested as a possible tactic to lessen these negative consequences [6].

β-Lactams are four-membered nitrogen-containing heterocycles that have long been of interest in pharmaceutical chemistry due to their importance as pharmacophores. They form the structural backbone of several bioactive compounds due to their chemical reactivity and biological versatility. Nitrogen-based heterocycles are particularly significant in medicinal chemistry, as they readily form hydrogen bonds with biological targets [7].

Numerous studies have demonstrated that 2-azetidinone derivatives exhibit diverse pharmacological activities, including antimicrobial, antifungal, antihistaminic, anti-inflammatory, antiviral, antioxidant, and anticancer properties [8]. Furthermore, 2-azetidinone scaffolds are incorporated into various clinically essential drugs, which exhibit potent antibacterial, antitubercular, anti-inflammatory, and antiviral effects, including anti-HIV activity [9,10].

Recent research indicates that azetidinone (β-lactam) derivatives exhibit prominent anti-inflammatory effects, mainly by inhibiting COX-2 and reducing pro-inflammatory cytokines. These compounds effectively decreased edema in animal models of acute inflammation, highlighting their potential as candidates for further anti-inflammatory drug development [11].

Computer-aided drug design (CADD) is now an essential part of modern drug discovery. It enables the prediction of pharmacological effects and molecular interactions of organic compounds using computational techniques, including molecular docking, molecular dynamics simulations, and ADMET profiling. These approaches substantially reduce the time and expenses involved in experimental screening by pinpointing promising candidates that exhibit strong binding properties and suitable drug-like profiles [12].

This investigation was conducted to design, synthesize, and thoroughly evaluate a novel series of naproxen–azetidinone hybrids as prospective anti-inflammatory agents with improved selectivity towards COX-2. The study incorporated several complementary phases, including in vitro anti-inflammatory and COX inhibitory assays, molecular docking, molecular dynamics simulation, and MM/GBSA binding free energy analyses to elucidate critical binding interactions and affinity trends, as well as DFT-based frontier molecular orbital (HOMO–LUMO) studies to correlate electronic properties with biological activity. Additionally, pharmacokinetic and drug-likeness properties were predicted in silico to assess the compounds’ potential as COX-2-selective inhibitors.

## 2. Results

The following section summarizes the main experimental and computational findings. Results are presented clearly and concisely to highlight key trends and their relevance to the study objectives.

### 2.1. Chemistry

**Scheme 1:**
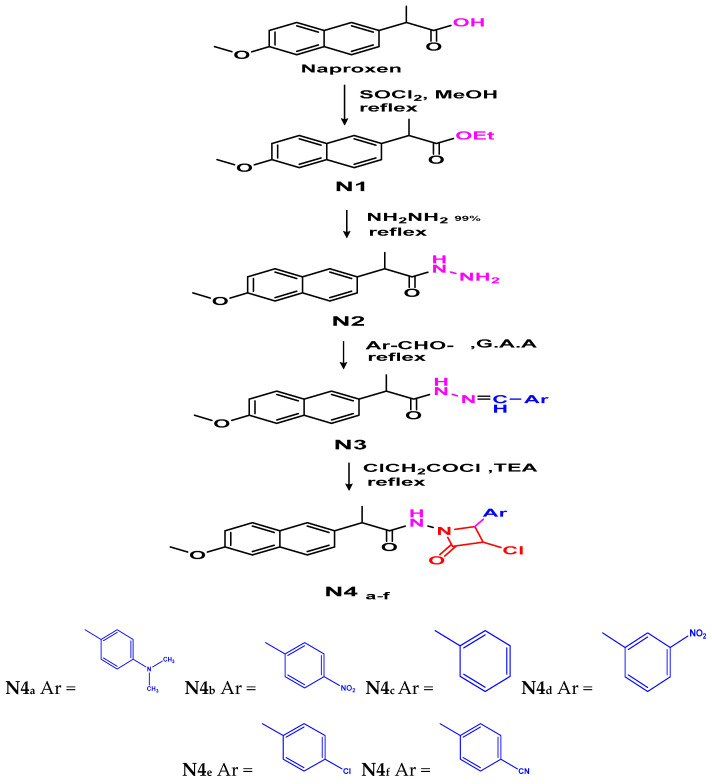
Synthesis of the intermediate and final compounds.

Starting with naproxen, the target derivatives (**N4_a–f_**) were created using a simple four-step procedure. The first stage involved the in situ production of the acyl chloride intermediate, followed by methanol’s nucleophilic attack, during which Naproxen reacted with SOCl_2_ and methanol to form its methyl ester. The ester was treated with 99% hydrazine in the next step, which transformed it into hydrazide (**N2**). To create hydrazone intermediates (**N3_a–f_**) by a nucleophilic addition–elimination process, the third step entailed the condensation of **N2** with substituted aromatic aldehydes. Ultimately, the strained β-lactam (2-azetidinone) ring was obtained by cyclizing these hydrazones with chloroacetyl chloride under basic conditions (TEA) by an intramolecular SN2 reaction. The appearance of a prominent carbonyl absorption band near approximately 1730 cm^−1^ in the IR spectrum confirmed the successful formation of the azetidinone carbonyl group. The target compounds were synthesized according to the procedures outlined in Scheme 1.

### 2.2. Biological Evaluation

In vivo, the acute anti-inflammatory effects of the final compounds (**N4_a_**–**N4_f_**) in egg white-induced paw edema were assessed. The reduction in paw thickness reflects the anti-inflammatory activity.

#### 2.2.1. Animals Methods

Albino rats (48 males), weighing 200 ± 10 g, were housed at the center under standardized conditions (12 h of light, 12 h of dark) for 7 days to acclimate in a temperature-controlled environment (22 ± 2 °C). They were kept at Tikrit University’s College of Veterinary Medicine. Before the experiment, they were housed in the laboratory for one week to reduce stress and establish stable baseline conditions. Subsequently, the animals were moved to the laboratory and divided into eight groups, each consisting of six rats, arranged as follows [13,14]. The experimental groups, corresponding doses, and % edema inhibition are summarized in Table 1.

Group A: Six rats served as controls and received the vehicle (50% *v*/*v* propylene glycol).

Group B: Six rats were treated with naproxen as a reference, at a dose of 10 mg/kg, suspended in propylene glycol.

Groups C–F: Each consisting of six rats, were treated with the final compounds (**N4_a_**–**N4_f_**) at doses specified below, also suspended in propylene glycol.

#### 2.2.2. Calculations for Dose Determination

The molecular weight of Naproxen = 230 g/mol. 10 mg/kg/230 = Dose/Molecular weight of the tested compound [15,16].

#### 2.2.3. Experimental Design

The anti-inflammatory activity of the tested compounds (**N4_a_**–**N4_f_**) was evaluated using the egg-white-induced edema model. Acute inflammation was initiated by injecting 0.05 mL of undiluted egg-white subcutaneously into the plantar surface of the left hind paw of rats, 30 min after administering the medicines or their carrier via intraperitoneal (i.p.) injection [17,18]. The paw thickness was measured by micrometer screw gauge (vernea) at seven-time intervals (0, 30, 60, 120, 180, 240, 300 min) after drug administration.

#### 2.2.4. Statistical Analysis

**Figure 1:**
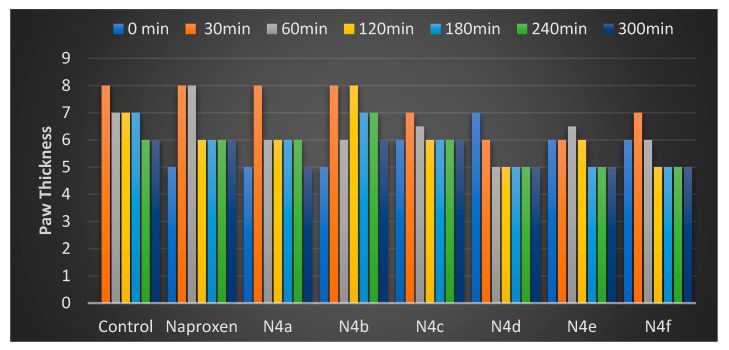
Diagram that shows the effect of propylene glycol, naproxen, and synthetic chemicals on the paw edema of rats induced by egg whites.

The data are expressed as the mean ± SEM. The results were analyzed for statistical significance using Student’s *t*-test (Two Sample Assuming Equal Variances) to compare mean values. Comparisons between groups were conducted using a two-factor ANOVA without replication. The statistical significance was discussed in terms of a *p*-value below 0.05. The anti-inflammatory effects of the resulting compounds were compared to those of naproxen and a 50% *v*/*v* solution of propylene glycol (control group). The investigated compounds and the reference medication had a significant decrease in paw edema compared to 50% *v*/*v* propylene glycol (Figure 1). All the tested drugs had a significant effect on reducing paw edema, with an onset of action of 120 min and high anti-inflammatory activity compared to naproxen (10 mg/kg). Also, none of the drugs lost their period of action during the study period, and the findings were statistically significant (*p* < 0.05) [19].

#### 2.2.5. Comparative Analysis

The resultant combination of theoretical and experimental results was similar and different in some ways. Docking and binding energy calculations consistently showed that **N4_c_**, **N4_d_**, and **N4_e_** were the most effective ligands, due to their strong binding affinities and stable interaction networks, compared to Naproxen. However, the trend was a bit different in the in vivo anti-inflammatory test. The compounds tested, including Naproxen, maintained their anti-inflammatory activities throughout the 300 min experiment, whereas the derivatives showed inconsistent effectiveness. The percentages of inhibition of **N4_d_**, **N4_e_**, and **N4_f_** were the highest (19.22%, 16.54% and 16.98%, respectively). This means that they had a significant biological impact. Moderate effects were observed in **N4_a_** (10.58%) and **N4_c_** (7.45%), with no considerable inhibition noted in **N4_b_**.

**N4_c_** had a stronger binding affinity in silico, but it still did not work well in tests. This means that pharmacokinetic or metabolic factors may affect how well drugs bind to each other.

**Figure 2:**
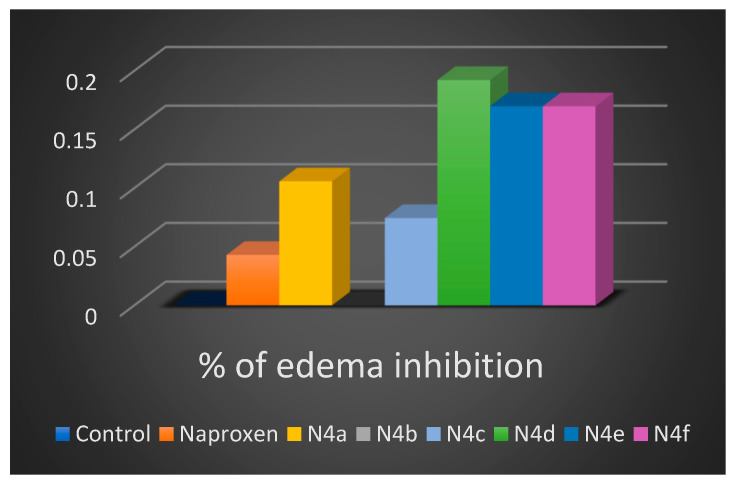
Effect of propylene glycol, naproxen, and synthesized compounds on egg white provoked paw edema in rats, showing percent edema inhibition.

These results demonstrate that computer predictions can provide valuable insights into the likelihood of binding, but further experiments are necessary to identify the most promising candidates for biological activity. Figure 2 shows that **N4_d_**, **N4_e_**, and **N4_f_** are the most effective derivatives compared to Naproxen.

### 2.3. In Silico Analysis

#### 2.3.1. Molecular Docking Simulation

This study aimed to analyze the inhibitory effects of newly synthesized compounds on COX-2 isoenzymes using computational docking. The crystallographic structures of the target protein COX-2 were obtained from the Protein Data Bank (PDB) database [PDB ID: 4M11]. Meloxicam, an established non-steroidal anti-inflammatory drug, was used as a reference for comparative analysis.

Computational docking analyses were conducted using Schrödinger software. Maestro (Schrödinger Release 2024-2, Schrödinger, LLC, New York, NY, USA). All investigated compounds were effective inhibitors, with binding free energies to the target protein COX-2 ranging from −11.93 to −9.72 Kcal/mol. The reference ligands meloxicam and naproxen had binding free energies of −9.30 Kcal/mol and −6.79 Kcal/mol, respectively, as shown in Table 2.

**Figure 3:**
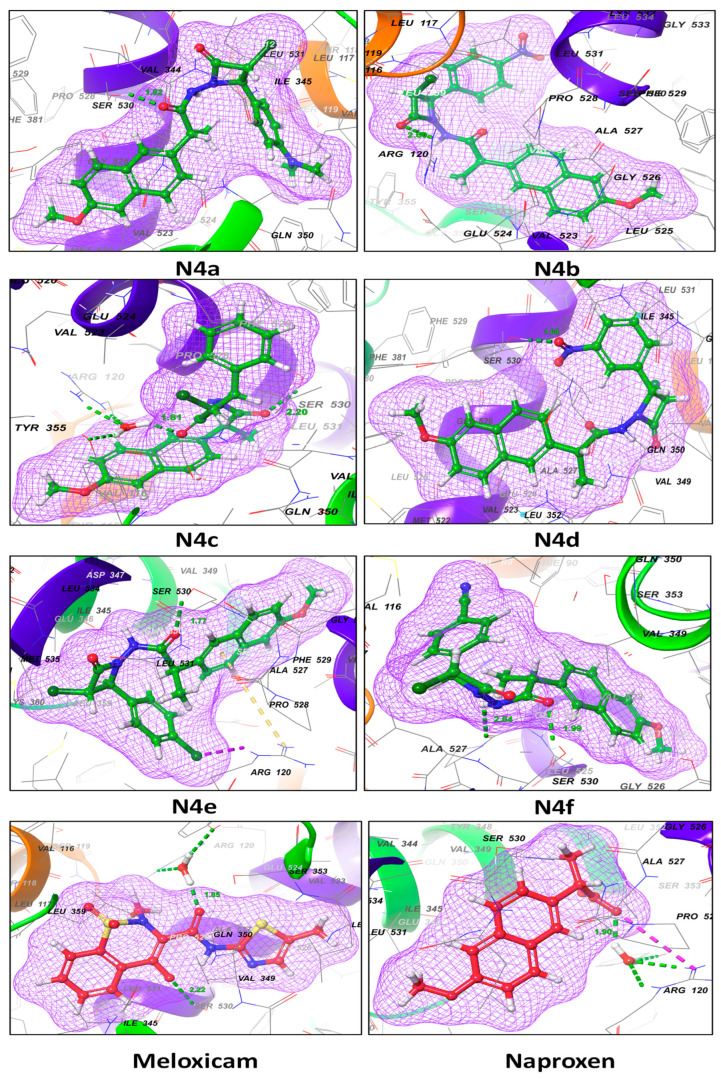
Compound interactions with COX-2’s active binding site in three dimensions with references.

**Figure 4:**
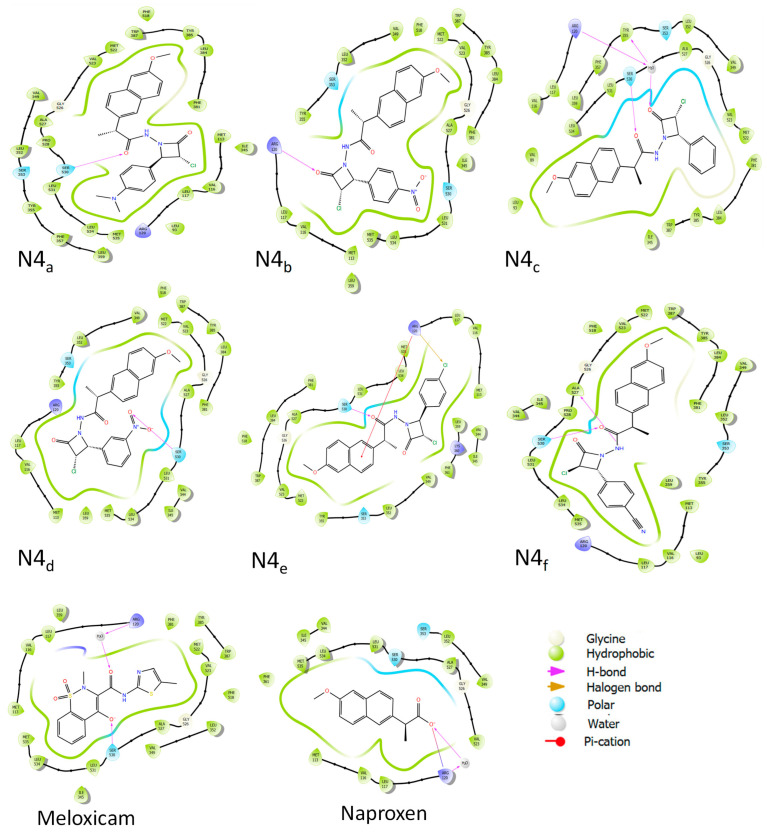
Compounds’ interactions with COX-2’s active binding site in two dimensions with references.

Docking simulations of the COX-2 target with the synthesized compounds demonstrated that intermolecular hydrogen bonds stabilized all the most stable energy complexes. Results showed that the compounds could access the substrate-binding region of the active site, with compound **N4_c_** achieving the highest score for COX-2, clearly demonstrating a strong link between in vivo and in silico studies. Figure 3 and Figure 4 display 3D and 2D images of the proposed compounds, along with their corresponding references.

Molecular docking against COX-2 also supported their potential activity, as assessed by MM/GBSA. **N4_c_** exhibited the strongest ΔG_bind_ (−62.27 kcal/mol) and high binding affinity (−10.92 kcal/mol), followed by **N4_d_** and **N4_e_**, which also showed stable binding patterns.

Key hydrogen bonds were observed with ARG120 and SER530, essential residues within the COX-2 active site. Additionally, **N4_b_** and **N4_d_** exhibited favorable ADMET profiles, accompanied by stable docking interactions, which represent increased selectivity and safety compared to naproxen and meloxicam, used as reference compounds.

The results indicate that **N4_c_**, **N4_d_**, and **N4_f_** are promising candidates for COX-2 inhibitors due to their favorable balance of binding affinity, stability, and pharmacokinetic properties.

#### 2.3.2. MM-GBSA Analysis

The Prime module of the Schrödinger molecular modeling package was used to calculate energy. The MM-GBSA analysis was employed to estimate the binding free energies of the proposed compounds and the co-crystallized ligand, naproxen, in their interaction with the COX-2 receptor. The MMGBSA free binding energy was used as the molecular coupling process. This method helps evaluate binding interactions and energetics between the ligand and the COX-2 receptor [20]. The positive free binding affinities of all the analogs proposed indicated a high possibility of inclusion in the COX-2 receptor. Docking studies provided an initial estimate of ligand-enzyme interactions, while MM/GBSA calculations offered a more accurate assessment of the binding free energies.

In the MM/GBSA binding free energy analysis, the binding affinities of synthesized derivatives to COX-2 were established to be considerably different. **N4_c_** had the highest binding energy of −62.27 kcal/mol, which is higher than that of meloxicam (−30.83 kcal/mol) and naproxen (−46.35 kcal/mol). Moreover, **N4_d_** (−49.46 kcal/mol) and **N4_e_** (−42.88 kcal/mol) exhibited better affinities than meloxicam, suggesting that substitutions enhance binding affinity. On the contrary, **N4_b_** (−32.92 kcal/mol) and **N4_f_** (−35.22 kcal/mol) had less improvement.

The following detailed examination of structural and physicochemical aspects enables a comprehensive understanding of why the binding affinity of **N4_c_** is significantly higher (MM/GBSA ΔG_bind_ = −62.27 kcal mol^−1^; docking score = −10.92 kcal mol^−1^). **N4_c_** is a key polar interactant of ARG120 and SER530, and also, it interacts with aromatic and hydrophobic residues in the active-site area (PHE381, TYR385, TRP387). These interactions, combined, enhance positive enthalpic contributions through van der Waals forces and hydrogen bonding. Its molecular weight is lower (408.1 Da) than that of other derivatives, and its lipophilicity (logP approximately 3.96) and TPSA (58.6 A^2^) are balanced. These characteristics enable the molecule to better fit into the binding pocket, lowering desolvation penalties and potentially reducing the strain on the ligand when binding. All these factors combined explain why **N4_c_** has a significantly lower ΔG_bind_.

These findings support the applicability of some structural changes, such as that of **N4_c_**, which is the most plausible alternative. Table 2 indicates that the compounds have a higher affinity for COX-2 than popular drugs. The docking fitness rating indicated that the majority of the derivatives predicted binding more effectively than the reference drugs, with **N4_a_**, **N4_d_**, and **N4_e_** achieving the highest scores. According to the agreement between scores and energies, **N4_c_**, **N4_d_**, and **N4_e_** are the most optimal. Among them, **N4_d_** is the most balanced profile, as it combines high binding energy, positive docking fitness, and stable molecular dynamics behavior, with conformational constraints, persistent contacts, and retention of protein secondary structure.

#### 2.3.3. Frontier Molecular Orbitals in Ligand–Receptor Interaction

One approach to studying ligand–receptor interaction is to model the approach of large molecules to the surface until binding or repulsion occurs. The concept of frontier molecular orbitals (FMOs), which encompasses the highest occupied molecular orbital (HOMO) and the lowest unoccupied molecular orbital (LUMO), helps us understand the electronic behavior of molecules. These orbitals help us understand how readily a compound can accept or lose electrons, which in turn influences its reactivity and its interaction with biological targets. Overall, molecules that have a smaller HOMO-LUMO gap are electronically flexible and require less energy to excite. This allows them to interact more with the active residues of the receptor in noncovalent ways such as hydrogen bonding, π–π stacking, and charge transfer [21].

Over the past few years, the integration of FMO analysis with molecular docking and binding energy calculations has proven to be a successful approach for explaining structure–activity relationships. This paper employs HOMO-LUMO analysis, along with docking, MM/GBSA, and molecular dynamics studies, to elucidate how electronic properties influence the binding of naproxen-azetidinone derivatives.

**Figure 5:**
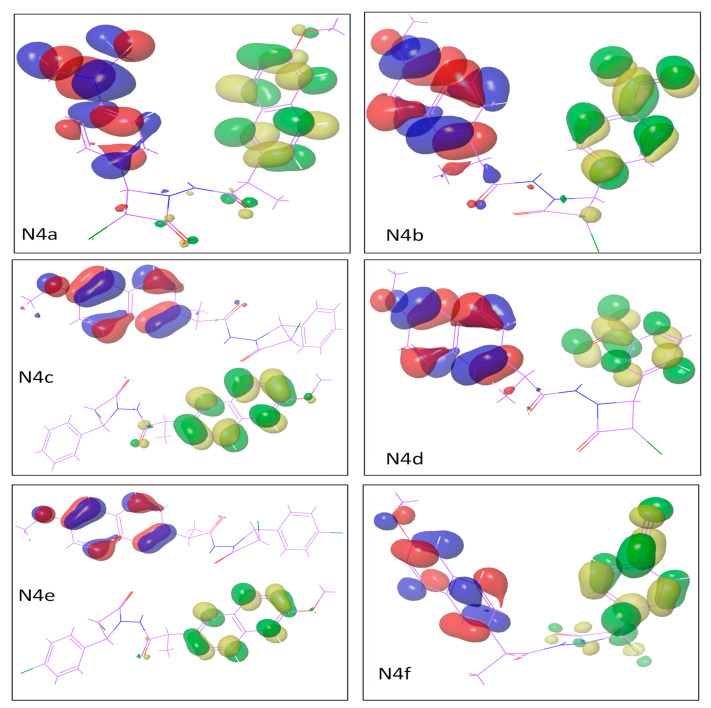
HOMO and LUMO of the designed compound (**N4_a_**–**N4_f_**). The HOMO orbital of every compound is displayed in the (**left panel**), and the LUMO orbital is shown in the (**right panel**). The positive and negative phases of the HOMO are indicated by the red and the blue lobes, and the green and the yellow lobes indicate the LUMO electron density distribution.

The outcomes of the DFT calculations (as shown in Table 3 and Figure 5) revealed that the HOMO-LUMO gaps of compounds **N4_b_** and **N4_c_** are the lowest (2.95 and 2.97 eV, respectively). This implies that they are highly polarizable and stand a higher possibility of charge transfer. The fact that the energy gap (ΔE) and the computed MM/GBSA binding energy (ΔG_bind_, ≈0.17) were weakly correlated implies that the electronic component alone cannot be used to explain the binding affinity fully.

One can visualize the molecular orbitals and gain an understanding. The MO density of both HOMO and LUMO of compound **N4_c_** is distributed uniformly in the aromatic region and the polar head group. This configuration enables π–π stacking and hydrophobic contacts to co-occur with residues such as Phe381, Tyr385, and Trp387. It also allows the polar interactions with ARG120 and SER530. This type of two-fold interaction increases favorable enthalpies and reduces the desolvation and strain energy. That is why its complex is much more stable (ΔG_bind_ = −62.27 kcal·mol^−1^).

Conversely, other derivatives, such as **N4_d_** and **N4_e_**, lack the same overlap between the donor and acceptor orbitals. **N4_b_** also has a small ΔE; however, its orientation within the binding pocket is less favorable. Moreover, the pattern of HOMO and LUMO levels provides a good indication of how ligands can bind to the COX-2 active site. The more energetic compounds, **N4_b_** and **N4_c_**, are more likely to participate in the electron donation process, and they can engage in the π–π and π-cation interactions with aromatic residues (PHE381, TYR385, TRP387). At the same time, the decreased LUMO levels of these compounds increase their ability to accept electron density of polar residues such as ARG120 and SER530, which are major contributors to the stability of the hydrogen-bonding network in the active pocket. This molecular orbital-amino acid complementarity accounts for the observed docking poses and MM/GBSA binding energies, indicating that electronic complementation is a crucial factor in the selective rejection of COX-2. Therefore, the visualization of FMO, combined with docking, provides a more accurate and more precise image of what makes **N4_c_** the most stable and reactive compound in the given series.

#### 2.3.4. Molecular Dynamics Simulation

**Figure 6:**
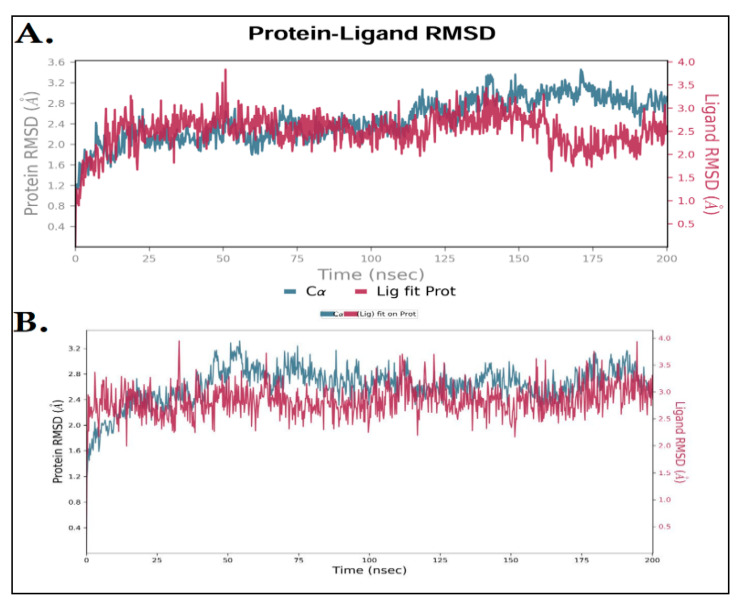
(**A**) compound **N4_d_** RMSD plot, (**B**) meloxicam RMSD plot.

Molecular dynamics (MD) simulations have been a key method for investigating the effects that ligands have on specific proteins, as the concept of conformational stability is significant in theoretical studies. This paper will review the stability of COX-2 in the presence of compound **N4_d_** and meloxicam after 200 nanoseconds. In analyzing the RMSD of the COX-2 backbone, we identified the effects of compound **N4_d_** on the protein’s structure, specifically focusing on conformational adjustments and ligand interactions. The modeling results give vital information on the physical changes of the protein. As shown in Figure 6A, the RMSD plot of compound **N4_d_** was consistently correlated with COX-2. The ligand RMSD oscillated around an approximate value of 2.0 Å, whereas the protein exhibited a fluctuation of 2.5 Å, which became stabilized after 200 ns during the MD simulation. The meloxicam RMSD graph indicated that the system had stabilized, with ligand RMSDs of 3.0 Å and 3.5 Å for protein RMSDs, as shown in Figure 6B.

**Figure 7:**
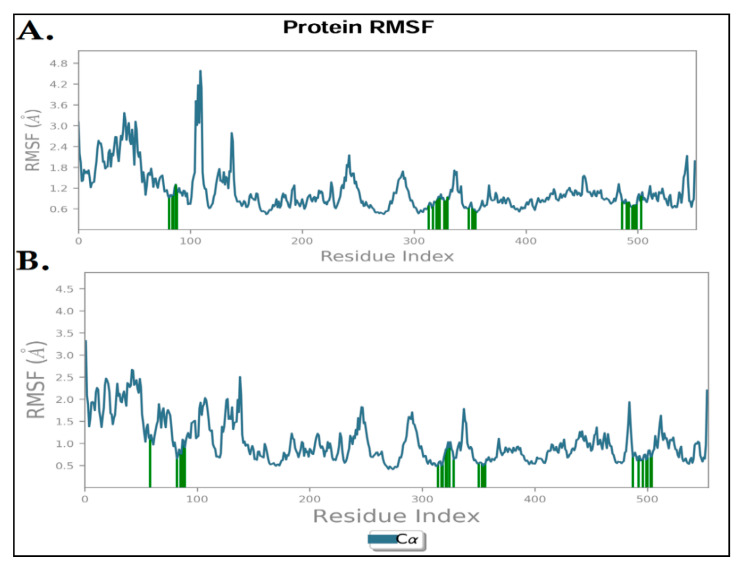
Protein fluctuations, as shown by RMSF, illustrate structural stability, while the green trace denotes regions interacting with the compounds (**A**) compound **N4_d_** RMSF, (**B**) meloxicam RMSF.

The RMSF of all binding residues for both compounds was below 1.5 Å, indicating stable interactions within the binding pocket throughout the MD simulation, as shown in Figure 7A,B.

**Figure 8:**
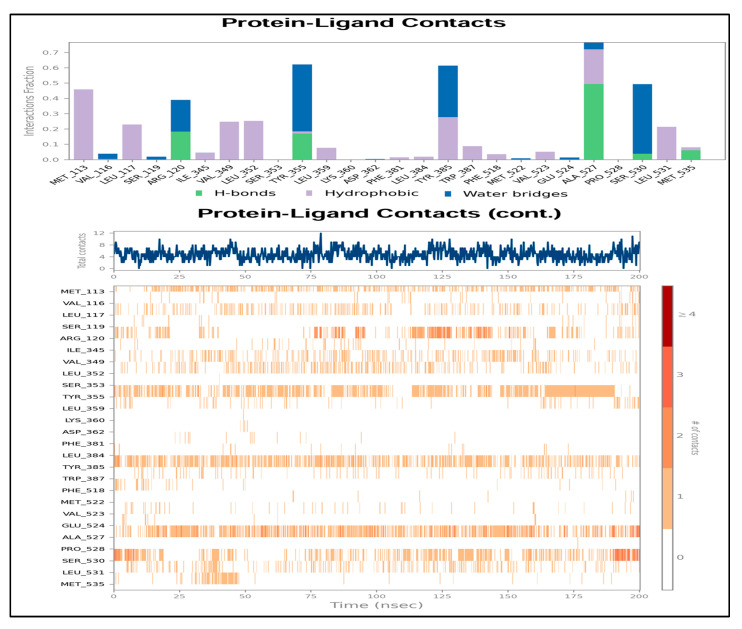
The interactions of COX-2 compound N4_d_ elucidate the proportion of binding interactions observed over a 200 ns Molecular Dynamics Simulation.

**Figure 9:**
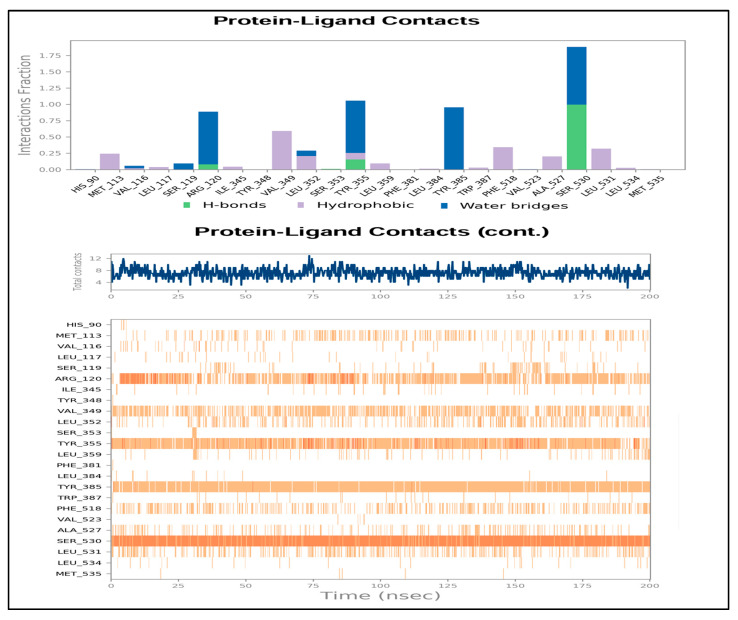
The contacts between COX-2 and meloxicam elucidate the proportion of binding interactions observed over a 200 ns molecular dynamics simulation.

During the simulation, compound **N4_d_** exhibited strong binding to COX-2 and interacted with most amino acids in its active site, as shown in Figure 8. It shared interactions with meloxicam, as shown in Figure 9, involving residues such as MET113, VAL116, ARG120, VAL349, TRY385, TYR355, ALA527, SER530, and LEU531 via hydrogen bonds, hydrophobic contacts, and water bridges.

This is in contrast to molecular dynamics (MD) simulations, which could be extended to over 200 ns or performed in many replicas to get improved statistical sampling; however, it was revealed that a single 200 ns simulation has been broadly accepted as being sufficient to define the conformational stability and binding dynamics of protein-ligand complexes [22,23]. These studies, which employed similar computational protocols, demonstrated that significant changes in structural or energetic behavior were not observed beyond 200 ns of simulation time, indicating that this timescale is sufficient to reach equilibrium and capture meaningful molecular interactions. Concurring with these results, the current experiment showed that RMSD and RMSF reached equilibrium and stabilized before 200 ns, indicating convergence of the simulated systems. Hence, the chosen time frame of 200 ns will provide a sufficient, scientifically valid window to examine the structural rigidity and binding stability of COX-2-ligand complexes.

#### 2.3.5. ADMET Evaluation

The candidates identified are likely the safest and most promising for further development, but may be more vulnerable to failure in later stages due to unfavorable ADMET properties. The pharmacokinetic characteristics—absorption, distribution, metabolism, excretion, and toxicity—of three naproxen derivatives were assessed using the freely accessible ADMETlab 2.0 tool (https://admetmesh.scbdd.com).

The chemical, pharmacokinetic, and physicochemical properties of these compounds are depicted in Table 4. The physicochemical profiling and ADMET of the synthesized naproxen–azetidinone analogs (**N4_a_**–**N4_f_**) provided valuable insights into their future drug-likeness and safety compared to naproxen and meloxicam.

The Lipinski’s Rule of Five (Ro5) offers a guideline for evaluating oral drug-likeness. It suggests that a compound should have a molecular weight under 500 Da, a calculated log P of 5 or less, no more than five hydrogen-bond donors, and no more than 10 hydrogen-bond acceptors. Compounds that violate more than one of these criteria are less likely to be suitable for oral administration and are less bioavailable [24]. The designed compounds were all in accordance with the rule of five established by Lipinski, implying that they possessed good oral bioavailability and favorable physicochemical characteristics. Their molecular weights ranged between 408.1 and 453.1 g/mol, which is a higher value compared to naproxen (230.09 g/mol), but still lower than 500 g/mol. This implies that they are of good size and can easily go through the membranes. The values of log P (3.7–4.7) indicate that the molecules are moderately lipophilic and can therefore pass through membranes without being too hydrophobic, which will make them less soluble and less likely to bioaccumulate. All the compounds had log S values of (−4.8 to −5.3), meaning that they were only moderately soluble, which is typical of NSAIDs. It is possible to improve it through various formulation approaches. The range of TPSA values (58–102 Å^2^) was within the optimal range (<140 Å^2^), indicating that the drug was well-absorbed.

The majority of the derivatives had low toxicity and lacked skin sensitization and reasonable LC50s. The Ames and carcinogenicity results were positive in all the compounds; however, occurrences of the same outcome are common in NSAID scaffolds and may be a result of metabolic activation, rather than genotoxicity. It is noteworthy that **N4_b_** and **N4_d_** were safe, and their predictions of LC50 were not toxic; there was no skin sensitization, and their physicochemical characteristics were good. These imply that they are less risky and more balanced alternatives.

**Figure 10:**
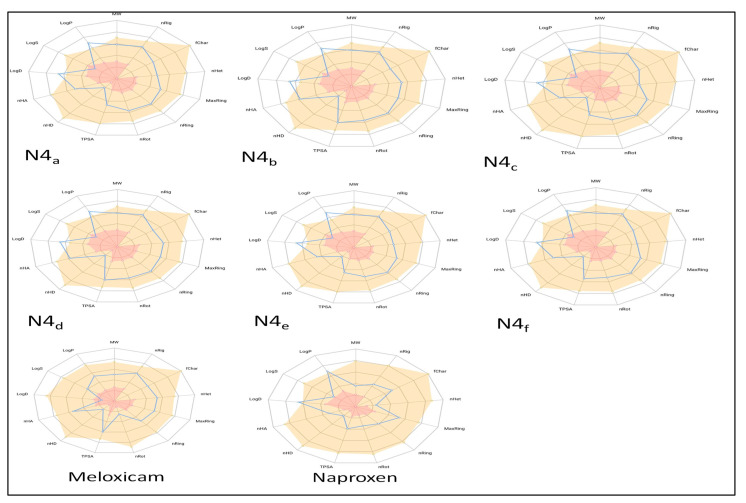
In silico ADMET profiling of compounds (**N4_a_**–**N4_f_**) and reference drugs (naproxen and meloxicam). Blue lines show predicted values, yellow areas indicate optimal ranges, and red areas show restricted zones.

hERG inhibition was predicted only for **N4_b_**, **N4_d_**, and naproxen, indicating limited cardiotoxicity risk. Most derivatives showed positive HIA values, suggesting good oral absorption. **N4_b_** and **N4_d_** have the best ADMET and physicochemical profiles, supporting their potential as COX-2 inhibitors. All summarized in Figure 10.

The promising pharmacokinetic and safety traits of the synthesized naproxen–azetidinone hybrids justified their molecular docking evaluation to confirm binding affinity and selectivity for COX-2.

## 3. Experimental Work

### 3.1. Materials and Methods

Chemicals used, unless stated otherwise, were bought from commercial suppliers and used as received. Naproxen was sourced from Sigma-Aldrich (Darmstadt, Germany). Melting points were measured with capillary tubes using a digital apparatus (STUART Scientific SMP30, Stone, UK) and are uncorrected.

Fourier-transform infrared (FT-IR) spectra were recorded on a Shimadzu 8400 spectrophotometer (Shimadzu, Kyoto, Japan) using KBr discs over the range 4000–400 cm^−1^. Proton nuclear magnetic resonance (^1^H NMR) spectra (Bruker, Billerica, MA, USA) were obtained on a 400 MHz spectrophotometer. Carbon nuclear magnetic resonance (^13^C NMR) spectra (Bruker, Billerica, MA, USA) were recorded on a 100 MHz instrument, using deuterated dimethyl sulfoxide (DMSO-*d*_6_) as the solvent and tetramethylsilane (TMS) as the internal standard.

### 3.2. Synthesis of Methyl (S)-2-(6-Methoxynaphthalen-2-yl) Propanoate *[**N1**]*


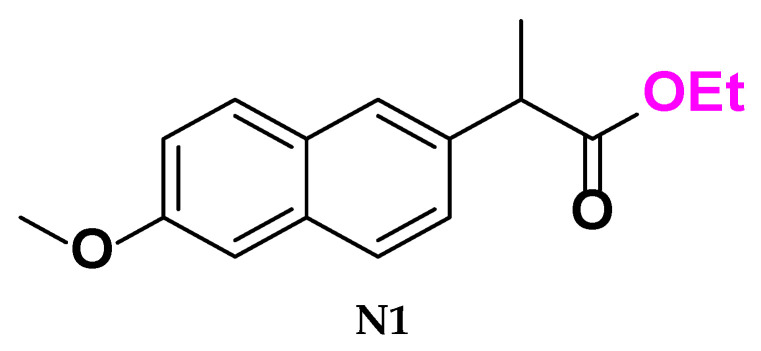


Dissolve 1 g (4.3 mmol) of [Naproxen] in 15 mL of absolute methanol until completely dissolved, and cool the mixture to 0–5 °C. Then add 0.32 mL (4.5 mmol) of thionyl chloride dropwise to the solution. Maintain the mixtures in a 40 °C water bath for 3 h, then reflux at 65 °C (the boiling point of the solvent) for 4 h. Finally, let the solutions cool and evaporate at room temperature.

25 mL of ice water was added to the resulting mixtures, which were then filtered and washed with a 10% *w*/*v* NaHCO_3_ solution (2 × 10 mL) to neutralize and remove any residual acidic impurities. This led to the formation of white crystals of the compound [**N1**]. Then, these products were purified by recrystallization from absolute ethanol to obtain pure products [25]. The reaction was monitored by thin-layer chromatography (TLC) on silica gel plates using an appropriate solvent system.

(**N1**) Physical properties:

White crystals (95% yield); m.p. 93 °C; FT-IR: 3057 cm^−1^ ν (C-H aliphatic), 1737 cm^−1^ ν (C=O of ester) and 1228 cm^−1^ ν(C-O). The figures are provided in the Supplementary File.

### 3.3. Synthesis of (S)-2-(6-Methoxynaphthalen-2-yl) Propane Hydrazide *[**N2**]*


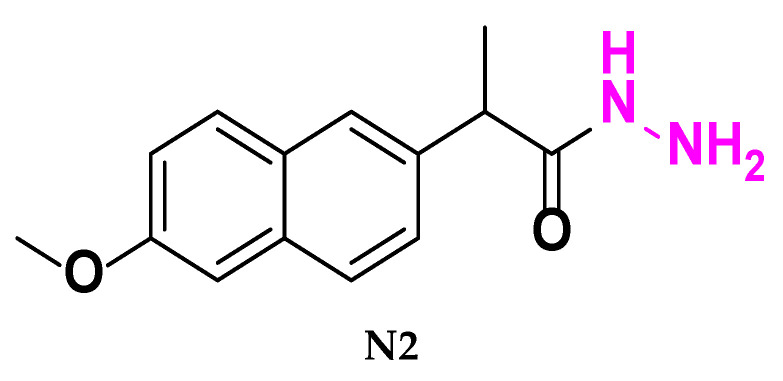


Using a 100 mL round-bottom flask, dissolve (0.244 g, 1 mmole) of compounds [**N1**] in 15 mL of absolute ethanol. Excess hydrazine hydrate (99%) was added to the solution (0.25 mL, 5 mmol), and then it was refluxed for 8 h. Upon cooling, the combination was precipitated using ice-distilled water and recrystallized from absolute ethanol. This process produced compound [**N2**], which appeared as a fluffy off-white powder [26]. The reaction was monitored by thin-layer chromatography (TLC) on silica gel plates using an appropriate solvent system.

(**N2**) Physical properties:

Off-White fine powder (60% yield); m.p. 114 °C; FT-IR: 3414–3308 cm^−1^ ν (N-H primary amine), 3250 cm^−1^ ν (N-H secondary amine), 3070 cm^−1^ ν (C-H aliphatic), and 1633 cm^−1^ ν (C=O amide). The figures are provided in the Supplementary File.

### 3.4. General Procedure for Synthesis of Schiff’s Bases Compounds *[**N3_a_**–**N3_f_**]*

To synthesize compounds [**N3_a_**–**N3_f_**], a solution of suitable aromatic aldehydes (0.1 g for powders or 0.1 mL for liquid aldehyde, one mmole) in 10 mL of absolute ethanol was prepared. The pH of the reaction mixture was maintained at a slightly acidic level (~pH 5–6) using a few drops of glacial acetic acid. Afterward, add 0.244 g (1 mmol) of compound [**N2**] to 15 mL of absolute ethanol. Then, add the mixture to the first mixture and reflux for 3 to 4 h at 75 to 80 °C.

After the reflux was complete, the mixtures were precipitated in a round-bottom flask. The precipitate was then separated by filtration, washed with cold deionized water, and recrystallized from absolute ethanol [26]. The reaction was monitored by thin-layer chromatography (TLC) on silica gel plates using an appropriate solvent system.

Physical properties (**N3_a_**):

Yellow to orange fluffy powder (60% yield); m.p. 182 °C; FT-IR: 3415 cm^−1^ ν (N-H secondary amine), 2920–2851 cm^−1^ ν (C-H aliphatic), 1651 cm^−1^ ν (C=O of amide) and 1604 cm^−1^ ν (C=N of imine), 1365 cm^−1^ ν (C-N of amide), and 1300 cm^−1^ ν (C-N of N(CH_3_)_2_). The figures are provided in the Supplementary File.

Physical properties (**N3_b_**):

Off-White fine powder (77% yield); m.p. 161 °C; FT-IR: 3180 cm^−1^ ν (N-H secondary amine), 2997–2841 cm^−1^ ν (C-H aliphatic), 1658 cm^−1^ ν (C=O of amide) and 1604 cm^−1^ ν (C=N of imine), 1564 cm^−1^ ν (N-O asymmetric of NO_2_), 1340 cm^−1^ ν (N-O symmetric of NO_2_). The figures are provided in the Supplementary File.

Physical properties (**N3_c_**):

White powder (80% yield); m.p. 157 °C; FT-IR: 3415–3236 cm^−1^ ν (N-H secondary amine), 2962–2929 cm^−1^ ν (C-H aliphatic), 1653 cm^−1^ ν (C=O of amide) and 1604 cm^−1^ ν (C=N of imine). The figures are provided in the Supplementary File.

Physical properties (**N3_d_**):

Off-White fine powder (76% yield); m.p. 153 °C; FT-IR: 3456 cm^−1^ ν (N-H secondary amine), 3007–2902 cm^−1^ ν (C-H aliphatic), 1658 cm^−1^ ν (C=O of amide) and 1604 cm^−1^ ν (C=N of imine), 1531 cm^−1^ ν (N-O asymmetric of NO_2_), 1344 cm^−1^ ν (N-O symmetric of NO_2_). The figures are provided in the Supplementary File.

Physical properties (**N3_e_**):

Off-White fine powder (58% yield); m.p. 141 °C; FT-IR: 3186 cm^−1^ ν (N-H secondary amine), 2935–2904 cm^−1^ ν (C-H aliphatic), 1649 cm^−1^ ν (C=O of amide), 1602 cm^−1^ ν (C=N of imine), 883 cm^−1^ ν (C-Cl stretching vibration). The figures are provided in the Supplementary File.

Physical properties (**N3_f_**):

Off-White powder (60% yield); m.p. 179 °C; FT-IR: 3182 cm^−1^ ν (N-H secondary amine), 2997–2891 cm^−1^ ν (C-H aliphatic), 2220 cm^−1^ ν (C≡N), 1654 cm^−1^ ν (C=O of amide), and 1602 cm^−1^ ν (C=N of imine). The figures are provided in the Supplementary File.


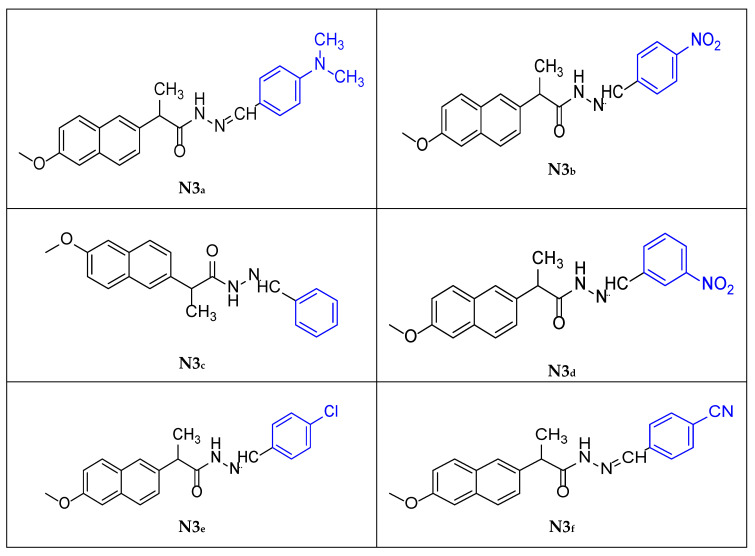


### 3.5. General Procedure for Synthesis of Azetidine-2-One Derivatives *[**N4_a_–N4_f_**]*

To synthesize [**N4_a_**–**N4_f_**], dissolve one mmole of the appropriate [**N3_a_**–**N3_f_**] in 15 mL of anhydrous tetrahydrofuran. Dropwise addition of (1.5 mmole, 0.169 g) of chloroacetyl chloride and (1 mmol, 0.101 g) of triethylamine over 15 min with stirring at 0–5 °C. The reaction mixture was stirred at room temperature for 3 h, and the solid (triethylamine hydrochloride) was removed. The solution was heated under reflux for 4 to 5 h at 65 °C, and then the solvent was evaporated at room temperature. The product was washed by using 10 mL of deionized water, filtered off, dried, and recrystallized from absolute ethanol [27].

Physical properties (**N4_a_**):

Brown liquid (50% yield); m.p. 189 °C; **FT-IR:** 3427 cm^−1^ ν (N-H secondary amine), 2974–2875 cm^−1^ ν (C-H aliphatic), 1730 cm^−1^ ν (C=O of 2-azetidinone ring), 1633 cm^−1^ ν (C=O stretching of amide), 1633 cm^−1^ ν (C-N of N(CH_3_)_2_), 821 cm^−1^ ν (C-Cl of the 2-azetidinone ring). The figures are provided in the Supplementary File. **^1^H-NMR:** 1.45 ppm (d, 3H, CH_3_), 2.93 ppm (s, 6H, N(CH_3_)_2_), 3.63 ppm (q, 1H, CH aliphatic) 4.71 ppm (s, 1H, CH-Cl-azetidine ring), 4.74 ppm (s, 1H, CH azetidine ring) and 7.11–7.76 ppm (d, 1H, 1H CH aromatic of naphthalene ring), 10.29 ppm (s, 1H, NH). The figures are provided in the Supplementary File. **^13^C-NMR:** 18.93 ppm (CH_3_ aliphatic), 66.42 ppm (CH-Cl azetidine ring), 67.02 ppm (CH- azetidine ring), 106.24–140.73 ppm (naphthalene ring), 124.45–144.67 ppm (aromatic ring attached to the NO_2_ group), 157.62 ppm (C=O of beta-lactam ring), 175.85 ppm (C=O of aliphatic chain). The figures are provided in the Supplementary File.

Physical properties (**N4_b_**):

Brown powder (66% yield); m.p. 178 °C; **FT-IR:** 3414 cm^−1^ ν (N-H secondary amine), 2922–2852 cm^−1^ ν (C-H aliphatic), 1732 cm^−1^ ν (C=O of 2-azetidinone ring) and 1658 cm^−1^ ν (C=O stretching of amide), 1606 cm^−1^ ν (C=N of imine), 1564 cm^−1^ ν (N-O asymmetric of NO_2_), 1340 cm^−1^ ν (N-O symmetric of NO_2_), 825 cm^−1^ ν (C-Cl of the 2-azetidinone ring). The figures are provided in the Supplementary File. **^1^H-NMR:** 1.48 ppm (d, 3H, CH_3_), 3.84 ppm (q, 1H, CH aliphatic) 4.79 ppm (s, 1H, CH-Cl azetidine ring), 4.84 ppm (s, 1H, CH azetidine ring) and 7.11–7.80 ppm (d, 1H, 1H CH aromatic of naphthalene ring), 7.51–8.15 ppm (d, 1H, CH aromatic ring with NO_2_ group), 11.57 ppm (s, 1H, NH). The figures are provided in the Supplementary File. **^13^C-NMR:** 18.93 ppm (CH_3_ aliphatic), 66.42 ppm (CH-Cl azetidine ring), 67.02 ppm (CH- azetidine ring), 106.24–140.73 ppm (naphthalene ring), 124.45–144.67 ppm (aromatic ring attached to the NO_2_ group), 157.62 ppm (C=O of beta-lactam ring), 175.85 ppm (C=O of aliphatic chain). The figures are provided in the Supplementary File.

Physical properties (**N4_c_**):

Brown liquid (56% yield); m.p. 186 °C; **FT-IR:** 3414 cm^−1^ ν (N-H secondary amine), 2956–2877 cm^−1^ ν (C-H aliphatic), 1730 cm^−1^ ν (C=O of 2-azetidinone ring), 1647 cm^−1^ ν (C=O stretching of amide), 1608 cm^−1^ ν (C=N of imine), 853 cm^−1^ ν (C-Cl of the 2-azetidinone ring). The figures are provided in the Supplementary File. **^1^H-NMR:** 1.44 ppm (d, 3H, CH_3_), 3.62 ppm (s, 1H, CH aliphatic) 4.19 ppm (s, 1H, CH-Cl-azetidine ring), 4.29 ppm (s, 1H, CH azetidine ring) and 7.19–7.78 ppm (d, 1H, 1H CH aromatic of naphthalene ring), 9.97 ppm (s, 1H, NH). The figures are provided in the Supplementary File. **^13^C-NMR:** 18.18 ppm (CH_3_ aliphatic), 63.11 ppm (CH-Cl azetidine ring), 67.55 ppm (CH- azetidine ring), 117.39–135.20 ppm (naphthalene ring), 128.73–133.52 ppm (benzene ring), 157.45 ppm (C=O of beta-lactam ring), 179.77 ppm (C=O of aliphatic chain). The figures are provided in the Supplementary File.

Physical properties (**N4_d_**):

Dark yellow powder (72% yield); m.p. 205 °C; **FT-IR:** 3414 cm^−1^ ν (N-H secondary amine), 3174 cm^−1^ ν (C-H aromatic), 2924–2852 cm^−1^ ν (C-H aliphatic), 1743 cm^−1^ ν (C=O of 2-azetidinone ring) and 1656 cm^−1^ ν (C=O stretching of amide), 1570 cm^−1^ ν (N-O asymmetric of NO_2_), 1344 cm^−1^ ν (N-O symmetric of NO_2_), 854 cm^−1^ ν (C-Cl of the 2-azetidinone ring). The figures are provided in the Supplementary File. **^1^H-NMR:** 1.47 ppm (d, 3H, CH_3_), 3.84 ppm (s, 1H, CH aliphatic) 4.12 ppm (s, 1H, CH-Cl azetidine ring), 4.29 ppm (s, 1H, CH azetidine ring) and 7.10–7.71 ppm (d, 1H, 1H CH aromatic of naphthalene ring), 7.50–8.7 ppm (d, 1H, CH aromatic ring with NO_2_ group), 11.37 ppm (s, 1H, NH). The figures are provided in the Supplementary File. **^13^C-NMR:** 18.90 ppm (CH_3_ aliphatic), 74.00 ppm (CH-Cl azetidine ring), 74.94 ppm (CH- azetidine ring), 106.19–137.53 ppm (naphthalene ring), 125.92–145.75 ppm (aromatic ring attached to the NO_2_ group), 165.16 ppm (C=O of beta-lactam ring), 175.53 ppm (C=O of aliphatic chain). The figures are provided in the Supplementary File.

Physical properties (**N4_e_**):

Brown powder (45% yield); m.p. 178 °C; **FT-IR:** 3180 cm^−1^ ν (N-H secondary amine), 2927–2852 cm^−1^ ν (C-H aliphatic), 1734 cm^−1^ ν (C=O of 2-azetidinone ring) and 1649 cm^−1^ ν (C=O stretching of amide), 1602 cm^−1^ ν (C=N of imine), 848 cm^−1^ ν (C-Cl in para position), 783 cm^−1^ ν (C-Cl of the 2-azetidinone ring). The figures are provided in the Supplementary File. **^1^H-NMR:** 1.47 ppm (d, 3H, CH_3_), 3.83 ppm (s, 1H, CH aliphatic) 4.76 ppm (s, 1H, CH-Cl azetidine ring), 4.87 ppm (s, 1H, CH azetidine ring) and 7.10–7.91 ppm (d, 1H, 1H CH aromatic of naphthalene ring), 7.48–7.51 ppm (d, 1H, CH aromatic ring with Cl group), 11.51 ppm (s, 1H, NH). The figures are provided in the Supplementary File. **^13^C-NMR:** 18.95 ppm (CH_3_ aliphatic), 65.21 ppm (CH-Cl azetidine ring), 65.65 ppm (CH- azetidine ring), 106.25–157.52 ppm (naphthalene ring), 128.88–133.33 ppm (aromatic ring attached to the Cl group), 157.61 ppm (C=O of beta-lactam ring), 175.68 ppm (C=O of aliphatic chain). The figures are provided in the Supplementary File.

Physical properties (**N4_f_**):

Brown powder (46% yield); m.p. 180 °C; **FT-IR:** 3414 cm^−1^ ν (N-H secondary amine), 3180 (C-H Aromatic), 2995–2891 cm^−1^ ν (C-H aliphatic), 2220 cm^−1^ νC≡N, 1660 cm^−1^ ν (C=O of 2-azetidinone ring), 1602 cm^−1^ ν (C=O stretching of amide), and 854 cm^−1^ ν (C-Cl of the 2-azetidinone ring). The figures are provided in the Supplementary File. **^1^H-NMR:** 1.48 ppm (d, 3H, CH_3_), 3.82 ppm (s, 1H, CH aliphatic) 4.83 ppm (s, 1H, CH-Cl azetidine ring), 4.84 ppm (s, 1H, CH azetidine ring) and 7.13–7.79 ppm (d, 1H, 1H CH aromatic of naphthalene ring), 7.46–7.48 ppm (d, 1H, CH aromatic ring with CN group), 10.30 ppm (s, 1H, NH). The figures are provided in the Supplementary File. **^13^C-NMR:** 18.81 ppm (CH_3_ aliphatic), 60.38 ppm (CH-Cl azetidine ring), 65.96 ppm (CH- azetidine ring), 106.16–139.19 ppm (naphthalene ring), 106.16–136.94 ppm (aromatic ring attached to the CN group), 166.41 ppm (C=O of beta-lactam ring), 171.14 ppm (C=O of aliphatic chain). The figures are provided in the Supplementary File.


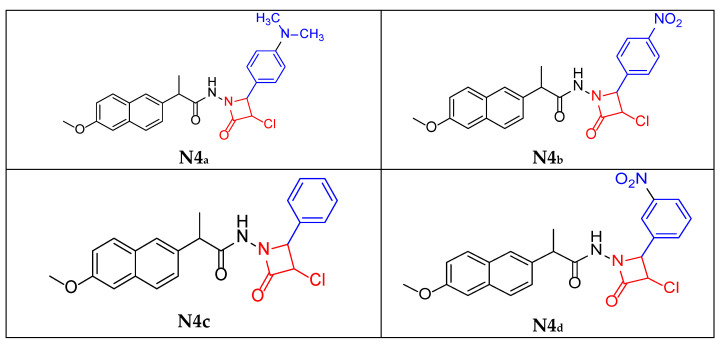
 
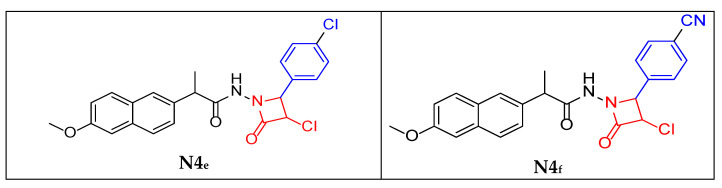


### 3.6. Computational Methods

In all computational investigations, the Schrödinger Maestro 2024-3 software package (Schrödinger LLC, New York, NY, USA) was used for molecular docking, MM/GBSA binding energy analysis, density functional theory (DFT) computations, molecular dynamics (MD) simulations, and ADMET predictions. To maintain constant energy parameters, the OPLS4 force field was employed throughout the calculation [28,29].

#### 3.6.1. Protein Preparation

The crystal structure of COX-2 (PDB ID: 4M11) was retrieved from the RCSB Protein Data Bank. Protein preparation was carried out using the Protein Preparation Wizard in Maestro (Schrödinger Release 2024-2, Schrödinger, LLC, New York, NY, USA), where bond orders were assigned, missing side chains and hydrogens were added, and protonation states were optimized at pH 7.4 using Epik version 5.8 (Schrödinger, LLC, New York, NY, USA). Water molecules beyond 3.0 Å from the active site were removed, and the structure was minimized under the OPLS4 force field [30,31].

#### 3.6.2. Ligand Preparation

The LigPrep module in Maestro (Schrödinger Release 2024-2, Schrödinger, LLC, New York, NY, USA) was used to generate three-dimensional conformations of the designed naproxen–azetidinone hybrids (**N4_a_**–**N4_f_**) after they were sketched in ChemDraw Professional version 22.0 (PerkinElmer Informatics, Waltham, MA, USA). The geometrical optimization of each ligand was performed using the OPLS4 force field at physiological pH (7.4) to obtain low-energy conformers. Before molecular docking, the stereochemistry and protonation states were carefully inspected and verified [32].

#### 3.6.3. Molecular Docking

Molecular docking was carried out using the Glide module (Extra Precision, XP) in Maestro (Schrödinger Release 2024-2, Schrödinger, LLC, New York, NY, USA). The receptor grid was generated around the COX-2 active site using the coordinates of the co-crystallized ligand. During docking, the receptor was kept rigid while the ligands were allowed complete flexibility. The best-scoring poses were selected based on the lowest GlideScore values and the presence of optimal hydrogen bonding, π–π stacking, and hydrophobic interactions within the binding pocket. Naproxen and meloxicam were used as reference ligands for comparison. Maestro visualization tools were used to analyze two- and three-dimensional interaction diagrams [33].

#### 3.6.4. MM/GBSA Binding Free Energy Calculation

The MM/GBSA binding free energy of the final results from the molecular docking was also improved using the Prime module of the Schrödinger Suite (Schrödinger Release 2024-2, Schrödinger, LLC, New York, NY, USA) [34]. Computation of ΔG bind using VSGB 2.1 solvation model in combination with the OPLS4 force field was performed as per the equation:

$$\Delta G_{bind}=G_{complex}-\left(G_{protein}+G_{ligand}\right)$$

where *G_complex_*, *G_protein_*, and *G_ligand_* are the free energies of the complex, free energy of the unbound receptor, and free energy of the unbound ligand, respectively. The remaining parameters remained at their default settings at the Schrödinger Prime, which were van der Waals, Coulombic, covalent, and solvation energy terms applied to every minimized structure [35].

#### 3.6.5. Density Functional Theory (DFT) Calculations

A program of the Schrödinger Suite was used to optimize the electronic structure and perform frontier molecular orbital (FMO) analysis, using Jaguar software version 12.2 (Schrödinger Release 2024-2, Schrödinger, LLC, New York, NY, USA). The values of HOMO, LUMO, and the energy gap were obtained using the B3LYP/6-31G(d,p) theoretical framework. To explain the charge distribution and electrical properties relevant to ligand–receptor interaction, the frontier orbitals were plotted [36].

#### 3.6.6. Molecular Dynamics (MD) Simulations

We utilized the Desmond module of the Schrödinger Suite (Schrödinger Release 2024-2, Schrödinger, LLC, New York, NY, USA) to investigate the stability of COX-2–ligand complexes over time. After being solvated in an orthorhombic TIP3P water box, the systems were neutralized by adding the right Na+ and Cl+ counterions. Molecular dynamics (MD) simulations were conducted using the Nose–Hoover thermostat and the Martyna–Tobias–Klein barostat under the NPT ensemble (300 K, 1 atm). After equilibration and system relaxation, a 200 ns production run was carried out. Monitoring hydrogen bond occupancy, root-mean-square deviation (RMSD), and root-mean-square fluctuation (RMSF) along the trajectory enabled analysis of conformational stability and interaction persistence [37].

#### 3.6.7. ADMET Prediction

Drug-likeness and in silico pharmacokinetic characteristics were assessed using the ADMETlab 2.0 online platform (http://www.admetmesh.scbdd.com) [38]. Several factors were analyzed, including logP, molecular weight, topological polar surface area (TPSA), Lipinski’s rule of five, and hydrogen bond donors and acceptors. To forecast oral bioavailability and potential pharmacokinetic behaviors, other physicochemical characteristics, such as solubility and gastrointestinal absorption, were examined.

## 4. Conclusions

A novel series of naproxen–azetidinone hybrids was successfully designed, synthesized, and characterized using FT-IR, ^1^H NMR, and ^13^C NMR analyses. In vivo tests showed that several derivatives, especially **N4_d_**, **N4_e_**, and **N4_f_**, had better inhibition of rat paw edema compared to naproxen. In silico ADMET profiling confirmed that all compounds adhere to Lipinski’s rule of five and exhibit good oral absorption, indicating strong drug-like properties.

Molecular docking revealed high binding affinities at the COX-2 active site, consistent with the in vivo results. MM/GBSA free-energy calculations supported this, with **N4_c_** showing the best binding energy (ΔG = −62.27 kcal/mol), outperforming meloxicam and naproxen. Molecular dynamics simulations confirmed that the ligand–COX-2 complexes remained stable.

Additionally, DFT (B3LYP/6-31G(d,p)) analysis of frontier molecular orbitals showed that **N4_c_** has a narrow HOMO–LUMO gap, suggesting higher electronic reactivity and better potential for charge transfer with COX-2 residues.

Overall, these data demonstrate that 2-azetidinone-modified naproxen hybrids can be considered a suitable choice for selective COX-2 inhibition, as they exhibit better pharmacokinetic and anti-inflammatory properties. Future studies should also look into the models of chronic inflammation, cytotoxicity, and pharmacodynamics to support their therapeutic effects.

## 5. Patents

A patent application related to this study is planned for future filing.

## Figures and Tables

**Table 1:** Final compounds, control, and reference drug, with their molecular weight, dose, time, and % of edema inhibition.

	Time	0 min	30 min	60 min	120 min	180 min	240 min	300 min	% of Edema Inhibition
Paw Thickness (mm)/*n* = 6	Control	6.02 ± 0.04	8.33 ± 0.06	7.54 ± 0.06	7.35 ± 0.07	7.15 ± 0.05	6.24 ± 0.04	6.20 ± 0.02	0
	Naproxen	5.82 ± 0.02	8.35 ± 0.02	8.07 ± 0.05	6.90 ± 0.03 *a	6.28 ± 0.04 *a	6.45 ± 0.03 *c	6.17 ± 0.04 *b	4.3%
	**N4_a_**	5.33 ± 0.03	8.39 ± 0.05	6.73 ± 0.04	6.49 ± 0.03 *c	6.89 ± 0.02 *c	5.419 ± 0.02 *c	6.29 ± 0.03 *b	10.58%
	**N4_b_**	5.42 ± 0.02	8.35 ± 0.02	6.17 ± 0.04	8.90 ± 0.04 *c	7.28 ± 0.04 *c	7.44 ± 0.03 *c	6.07 ± 0.04 *b	0
	**N4_c_**	6.24 ± 0.03	7.39 ± 0.03	6.97 ± 0.05	6.90 ± 0.04 *c	6.88 ± 0.02 *c	6.47 ± 0.03 *c	6.19 ± 0.02 *b	7.45%
	**N4_d_**	7.42 ± 0.02	6.35 ± 0.02	5.17 ± 0.04	5.90 ± 0.04 *c	5.28 ± 0.04 *c	5.44 ± 0.03 *c	5.07 ± 0.04 *b	19.22%
	**N4_e_**	6.42 ± 0.02	6.35 ± 0.02	6.17 ± 0.04	6.90 ± 0.04 *c	5.28 ± 0.04 *c	5.44 ± 0.03 *c	5.07 ± 0.04 *b	16.98%
	**N4_f_**	6.33 ± 0.03	7.39 ± 0.05	6.73 ± 0.04	5.49 ± 0.03 *c	5.89 ± 0.02 *c	5.419 ± 0.02 *c	5.29 ± 0.03 *b	16.98%

* Significantly, the difference compared to the control was statistically significant (*p* < 0.05). Data are shown as mean ± SEM of paw thickness in millimeters. ‘n’ refers to the number of animals. Time 0 marks the injection of naproxen-tested compounds or propylene glycol via i.p, while Time 30 indicates when egg white was injected to induce paw edema. Different letters (a, b, c) among the tested compounds denote significant differences (*p* < 0.05).

**Table 2:** Molecular docking and MMGBSA results of naproxen-azetidinone derivatives at the COX-2 active site.

No.	Ligand	3D Structure	Protein	Docking Score (Kcal/mol)	MM/GBSA ΔG_bind (kcal/mol, ±SE)	Interaction Type		
						H-Bond	Pi-Pi Stacking	Short Contact
1	**N4_a_**	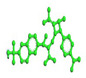	Murine cyclooxygenase-2 (PDB: ID: (4M11)	−11.93	−38.2 ± 0.6	SER530	-	LEU531, LEU534, MET535, ARG120, LEU117, VAL116, MET113, PHE381, TYR385, TRP387, MET522, VAL523, GLY526 *
2	**N4_b_**	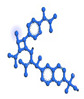		−9.72	−32.9 ± 0.5	ARG120	-	TYR355, LEU352, VAL349, MET522, VAL523, MET522, VAL523, GLY526 *, ALA527, LEU531, LEU534, MET535, MET113, VAL116, LEU117, SER353 *, SER530 *
3	**N4_c_**	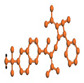		−10.92	−62.2 ± 0.7	ARG120, TYR355, SER530	-	VAL89, LEU93, ILE345, TRP387, TYR385, PHE381, GLY326 *, SER353 *
4	**N4_d_**	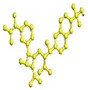		−10.59	−49.4 ± 0.4	SER530	-	ARG120, LEU117, VAL116, MET113, MET535, LEU534, LEU531, ALA527, GLY526 *, VAL523, MET522, VAL349, LEU352
5	**N4_e_**	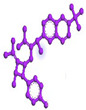		−10.57	−42.8 ± 0.6	SER530	ARG120	ARG120, TYR355, SER353 *, LEU352, VAL349, PHE361, LYS360, LEU359, MET113, MET535, LEU534, LEU531, ALA527, VAL523, MET522
6	**N4_f_**	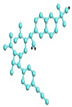		−11.53	−35.2 ± 0.5	-	ALA527, SER530	PHE381, LEU384, TYR385, TPR387, MET522, VAL523, GLY526 *, ALA527, SER530 *, LEU531, LEU534, MET535, ARG120, LEU117, VAL116, MET113, LEU359
7	Naproxen	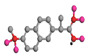		−6.79	−46.3 ± 0.4	-	ARG120	LEU117, VAL116, MET113, MET535, LEU534, GLY526 *, VAL523
8	Meloxicam	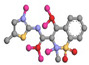		−9.30	−30.8 ± 0.5	SER530, ARG120	-	MET113, MET535, LEU534, LEU531, ALA527, GLY526 *, VAL523, MET522, ARG120

The asterisk (*) shows the type of interaction: green means glycine interaction, and blue means polar interaction with serine.

**Table 3:** Calculated the energy gap (ΔE), HOMO, and LUMO for designed derivatives.

Compound Code	HOMO (eV)	LUMO (eV)	ΔE (eV)
**N4_a_**	−5.13	−1.27	3.86
**N4_b_**	−5.67	−2.72	2.95
**N4_c_**	−5.61	−2.64	2.97
**N4_d_**	−5.62	−1.33	4.29
**N4_e_**	−5.63	−1.34	4.29
**N4_f_**	−5.67	−1.68	3.99

**Table 4:** Predicted ADMET profiles and physicochemical properties of naproxen derivatives (**N4_a_**–**N4_f_**) and reference drugs (naproxen and meloxicam).

No.	Ligand	Toxicity Risks					Physicochemical Properties							
		AMES Toxicity	Carcinogenicity	LC50 (Oral Rat)	Skin Sensitization	hERG Blockers	Molecular Weight	TPSA	Lipinski Rule	HBA	HBD	HIA	LogP	logS
1	**N4_a_**	(+)	(+)	(+)	(−)	(−)	451.1	61.8	(Accepted)	6	1	(+)	4.284	−5.29
2	**N4_b_**	(+)	(+)	(−)	(−)	(+)	453.1	101.7	(Accepted)	8	1	(+)	3.916	−4.84
3	**N4_c_**	(+)	(+)	(+)	(−)	(−)	408.1	58.6	(Accepted)	5	1	(−)	3.960	−4.98
4	**N4_d_**	(+)	(+)	(−)	(−)	(+)	453.1	101.7	(Accepted)	8	1	(+)	3.899	−4.81
5	**N4_e_**	(+)	(−)	(+)	(−)	(−)	442.0	58.6	(Accepted)	5	1	(+)	4.716	−5.20
6	**N4_f_**	(+)	(+)	(+)	(−)	(−)	433.1	82.43	(Accepted)	6	1	(−)	3.759	−5.01
7	Naproxen	(+)	(−)	(+)	(−)	(+)	230.0	46.5	(Accepted)	3	1	(+)	3.26	−3.85
8	Meloxicam	(−)	(+)	(−)	(−)	(−)	351.0	99.6	(Accepted)	5	0	(−)	1.68	−2.57

## Data Availability

All data supporting the findings of this study are available in the Supplementary Materials associated with this article.

## Supplementary Materials

The following supporting information can be downloaded at: https://www.mdpi.com/article/10.3390/molecules30224358/s1, Figures S1–S15: FT-IR spectrum of compounds; Figures S16–S21: Compounds’ ^1^H-NMR spectra; Figures S22–S27: Compounds ^13^C-NMR spectra.
