# Supplementary material for: Design, In Silico, and Experimental Evaluation of Novel Naproxen–Azetidinone Hybrids as Selective COX-2 Inhibitors
Source: Molecules. 2025 Nov 11;30(22):4358. doi: 10.3390/molecules30224358 (PMC12654640; doi:10.3390/molecules30224358)
Supplement: Supplementary file 1 [file molecules-30-04358-s001.zip › molecules-3968552-supplementary.pdf]

Design, In Silico, and Experimental Evaluation of Novel Naproxen–Azetidinone Hybrids as Selective COX-2 Inhibitors

Ayad K. Khan[1],*, Noor R. Mahmood[1], Mohammed A. Sahib[2]

[1] Department of Pharmaceutical Chemistry, College of Pharmacy, Mustansiriyah University, Baghdad, Iraq; (ayad@uomustansiriyah.edu.iq), (noor92@uomustansiriyah.edu.iq)
[2] Department of Pharmaceutical Chemistry, College of Pharmacy, University of Kerbala, Karbala, Iraq; (mohammed.a.sahib@uokerbala.edu.iq).
* Correspondence: ayad@uomustansiriyah.edu.iq.

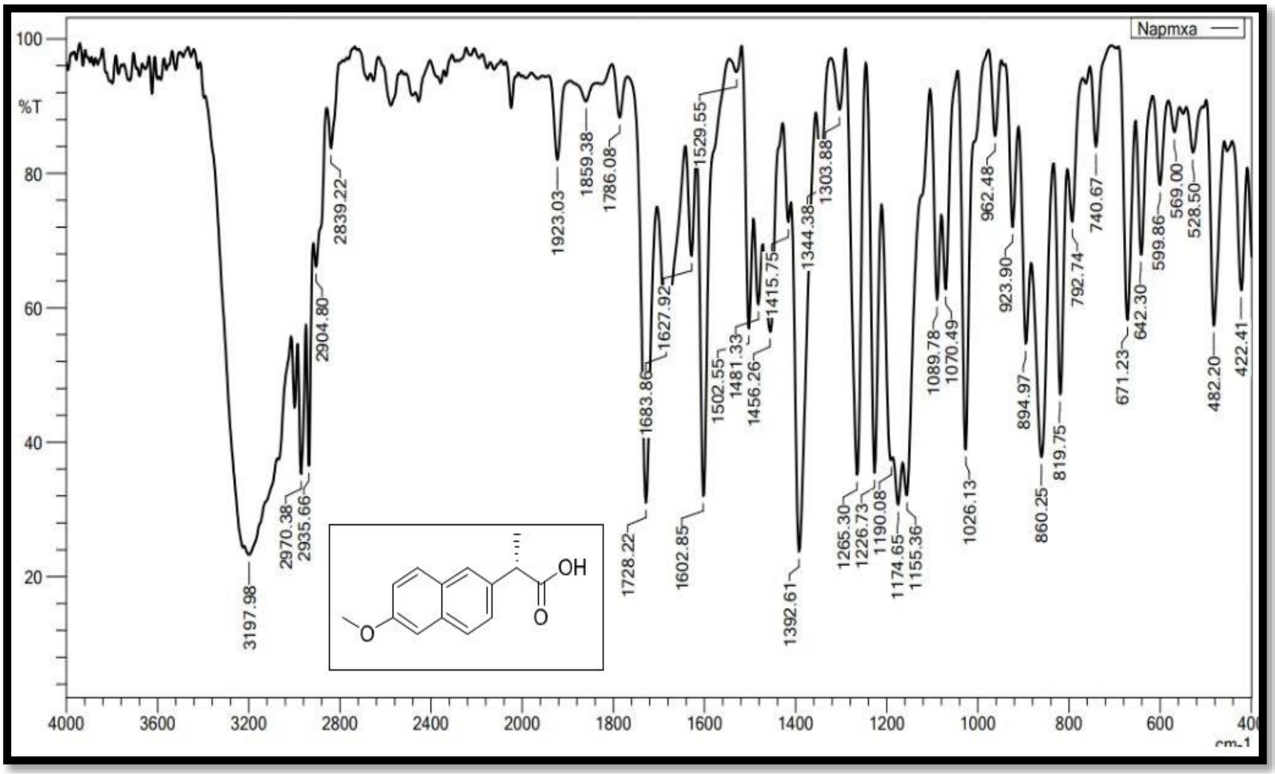

Figure S1: FT-IR spectrum of compound [Naproxen].

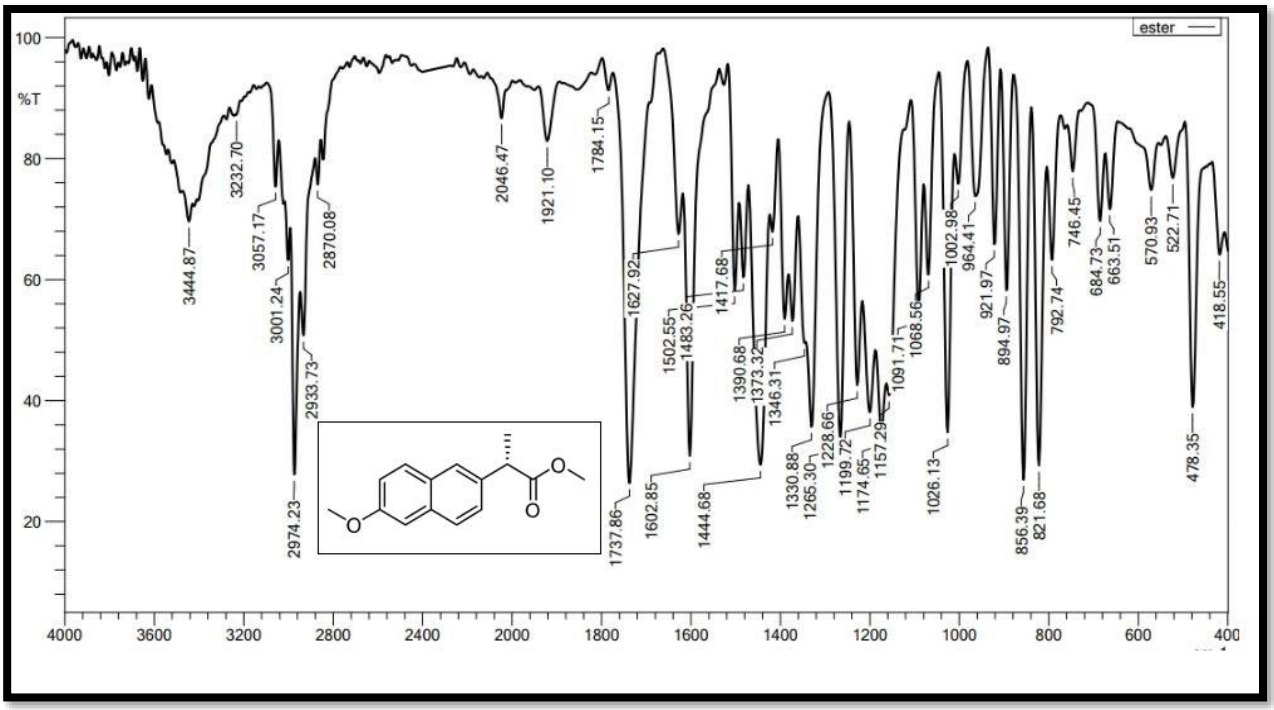

Figure S2: FT-IR spectrum of compound [N1].

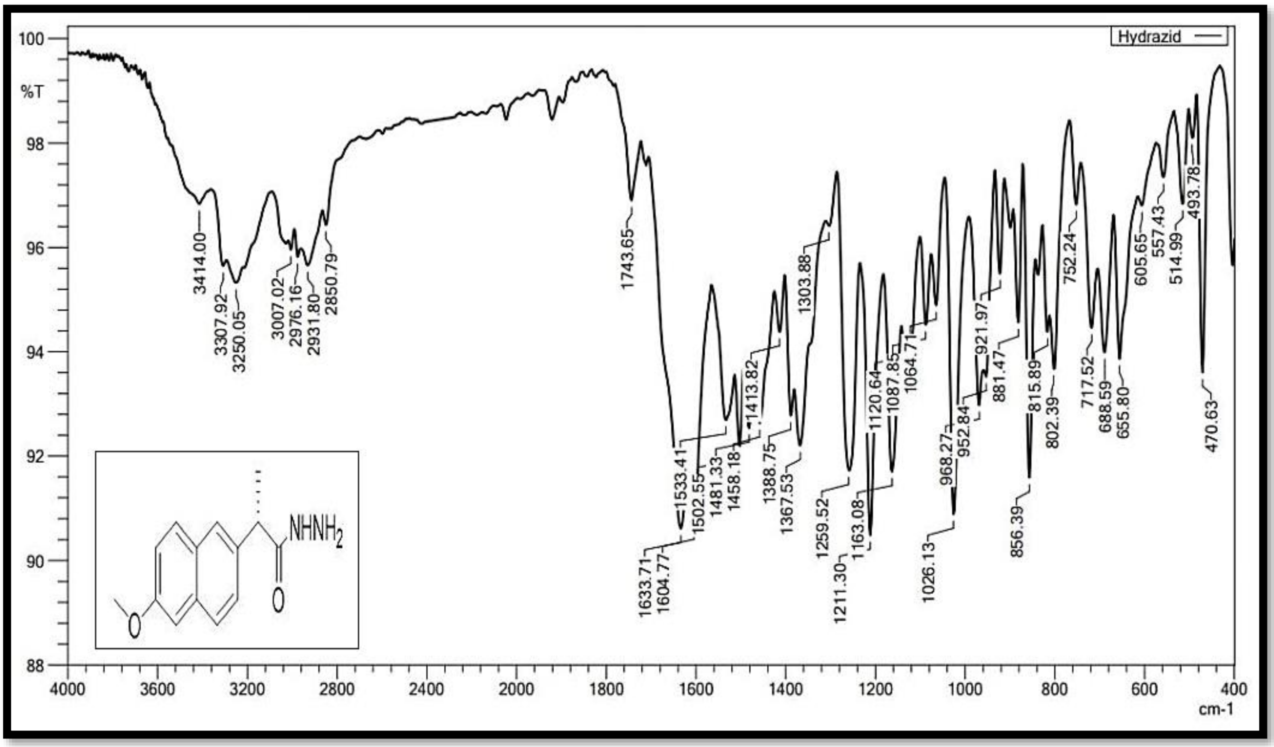

Figure S3: FT-IR spectrum of compound [N2].

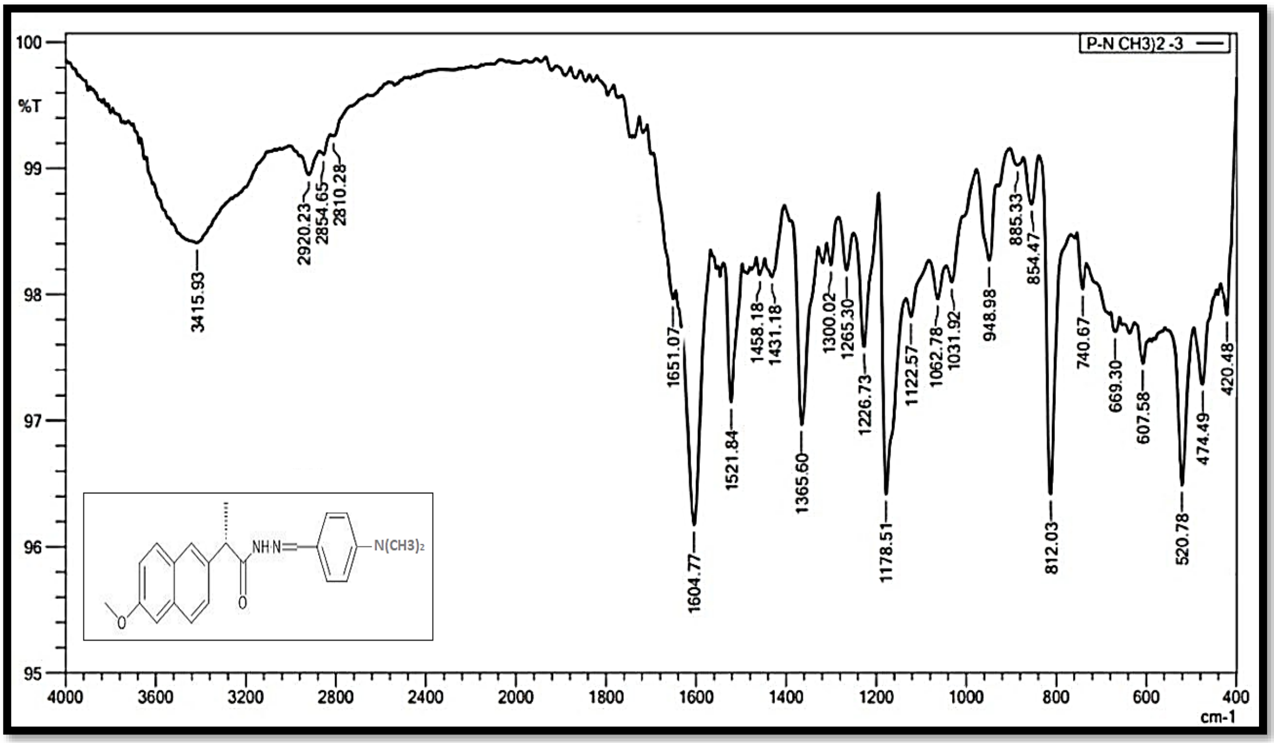

Figure S4: FT-IR spectrum of compound [N3a].

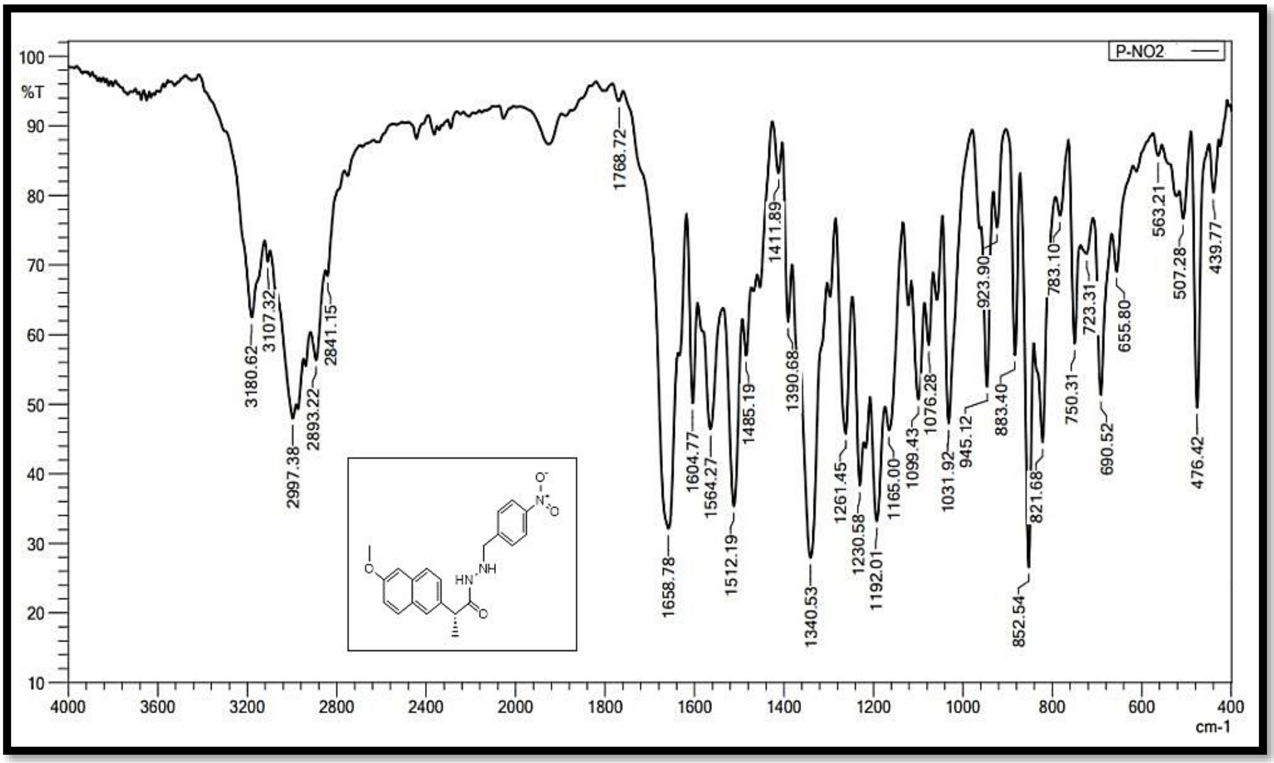

Figure S5: FT-IR spectrum of compound [N3b].

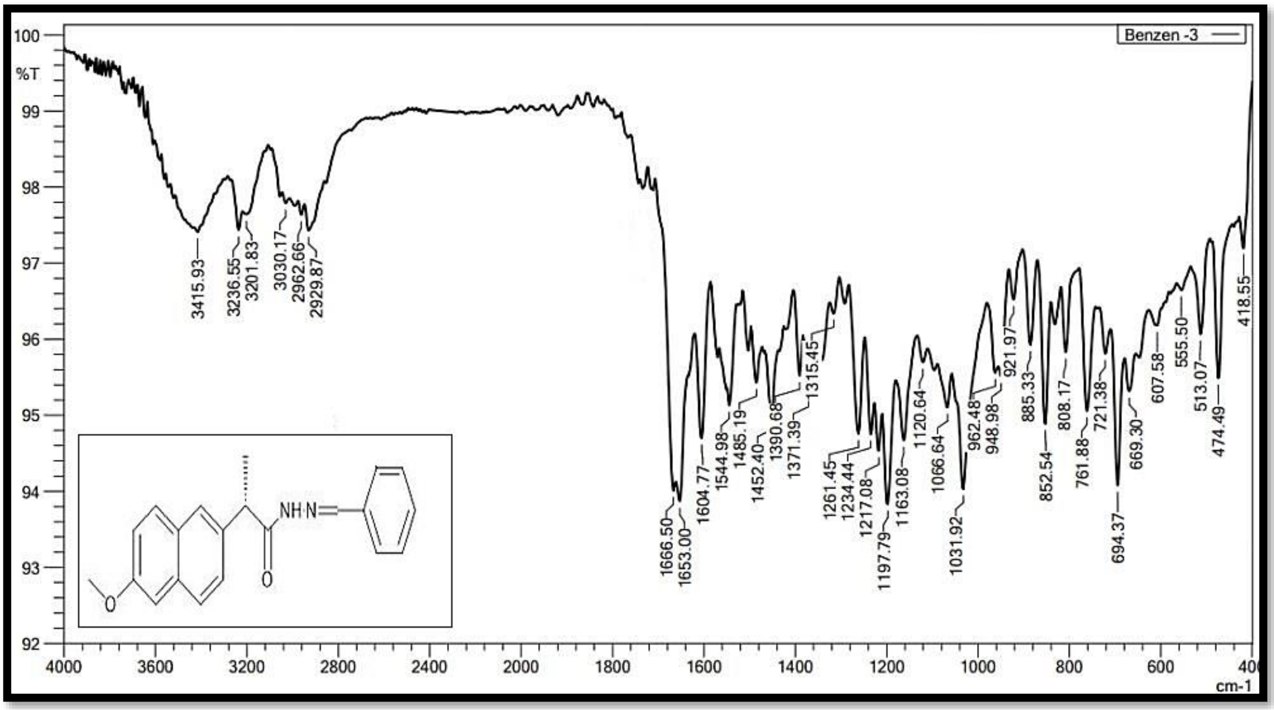

Figure S6: FT-IR spectrum of compound [N3c].

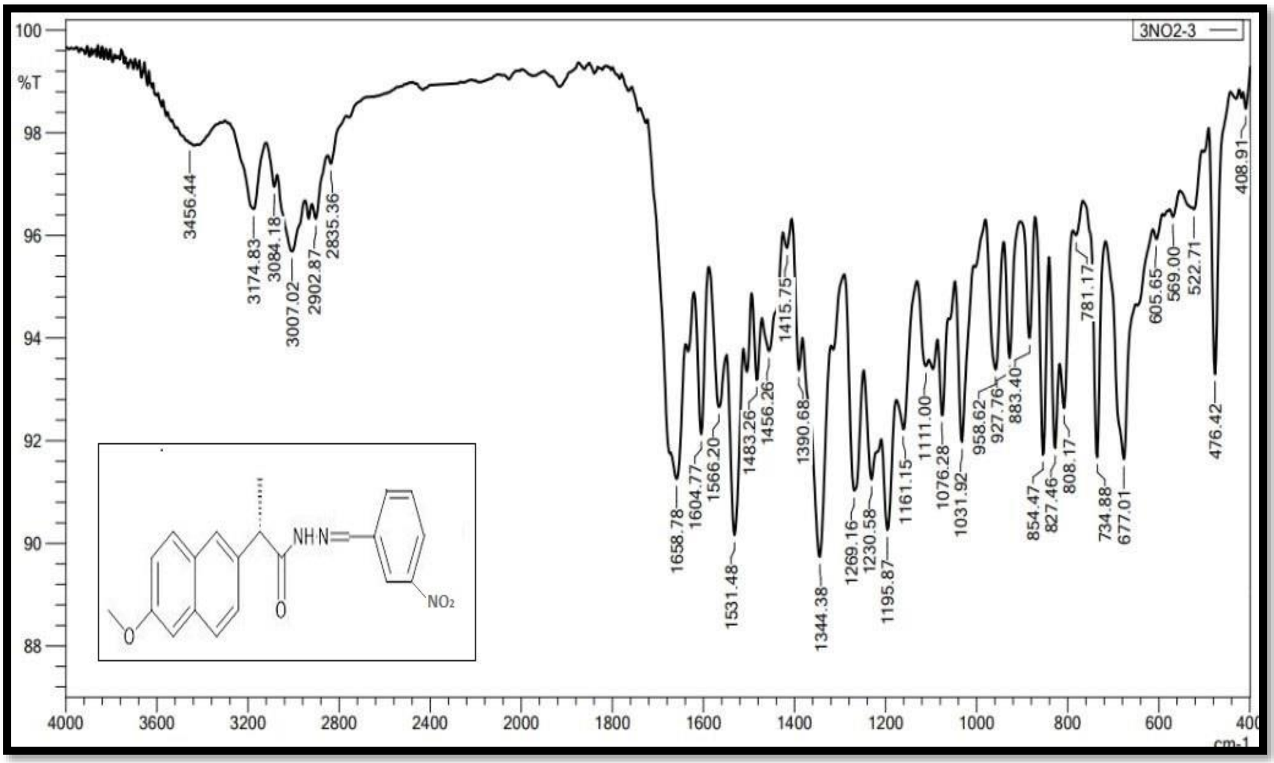

Figure S7: FT-IR spectrum of compound [N3d].

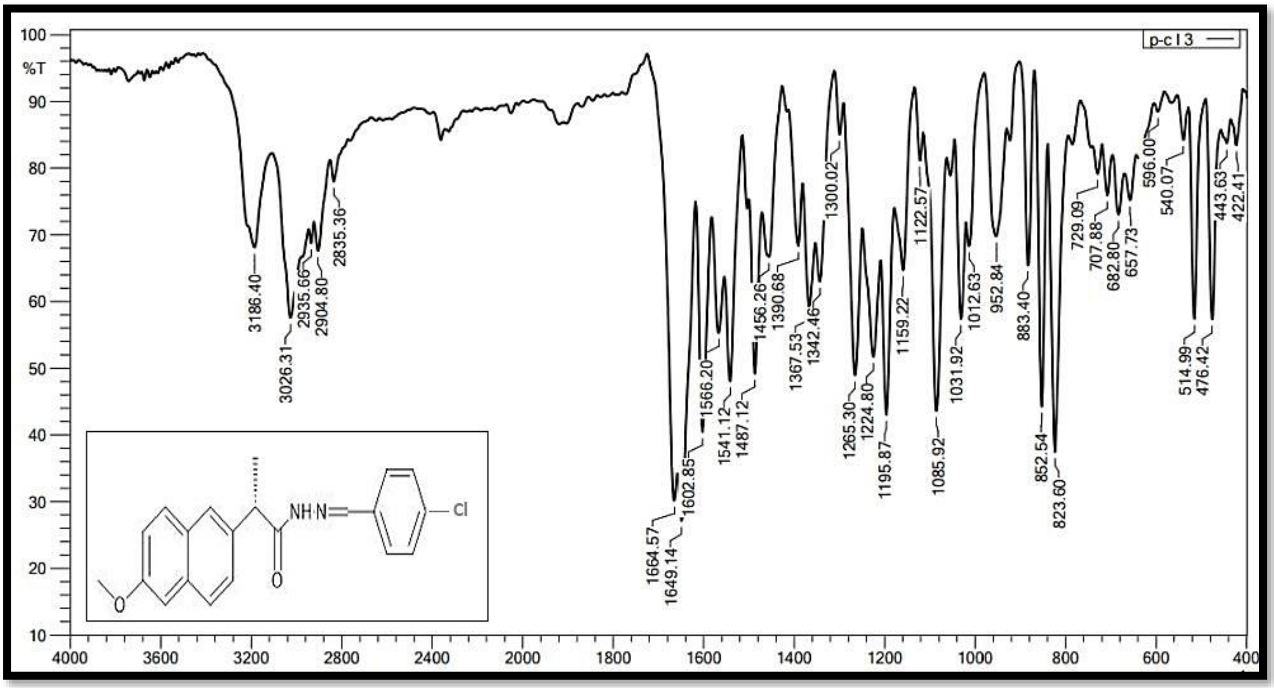

Figure S8: FT-IR spectrum of compound [$N3_e$].

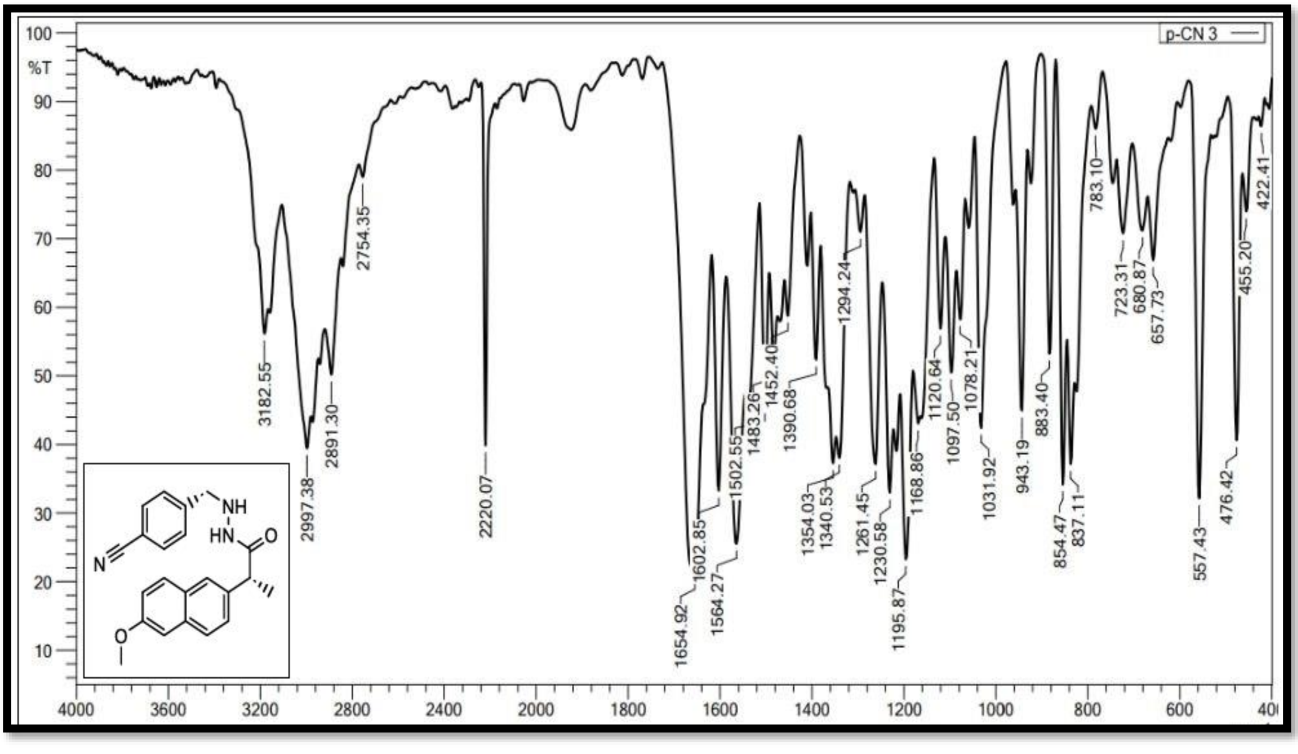

Figure S9: FT-IR spectrum of compound [$N3_f$].

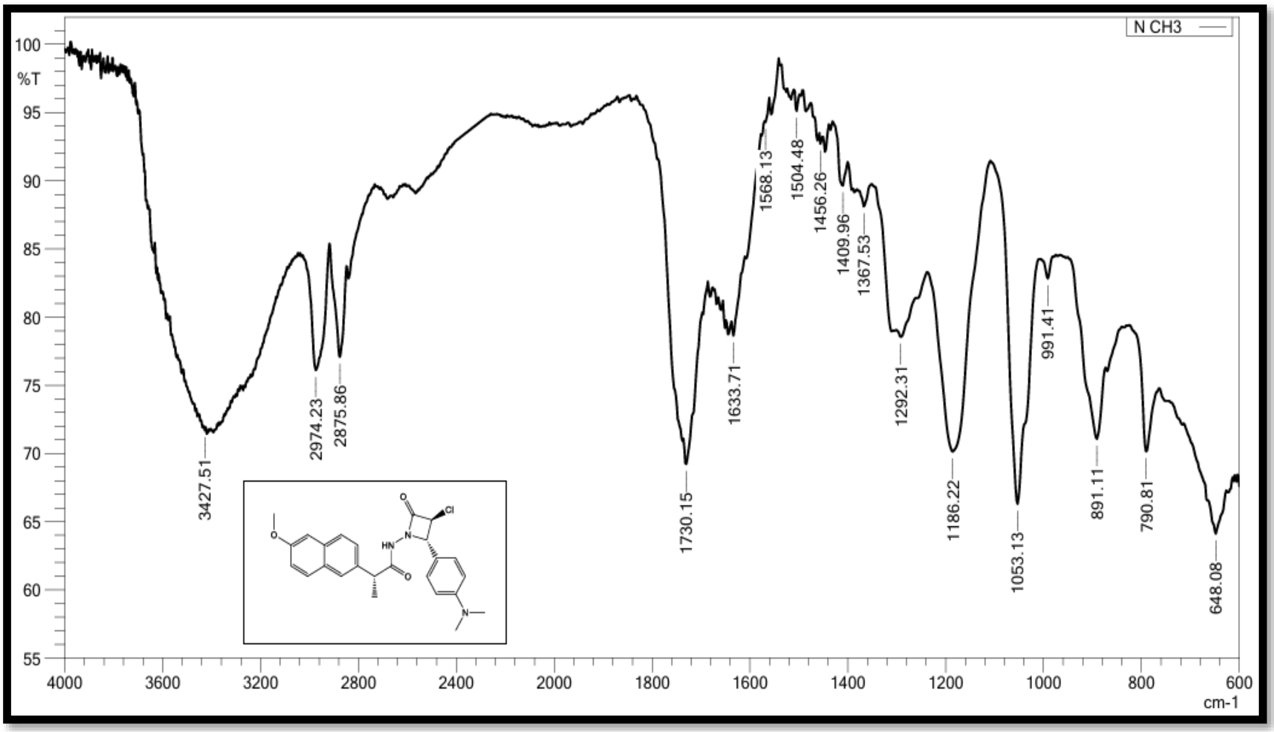

Figure S10: FT-IR spectrum of compound [N4a].

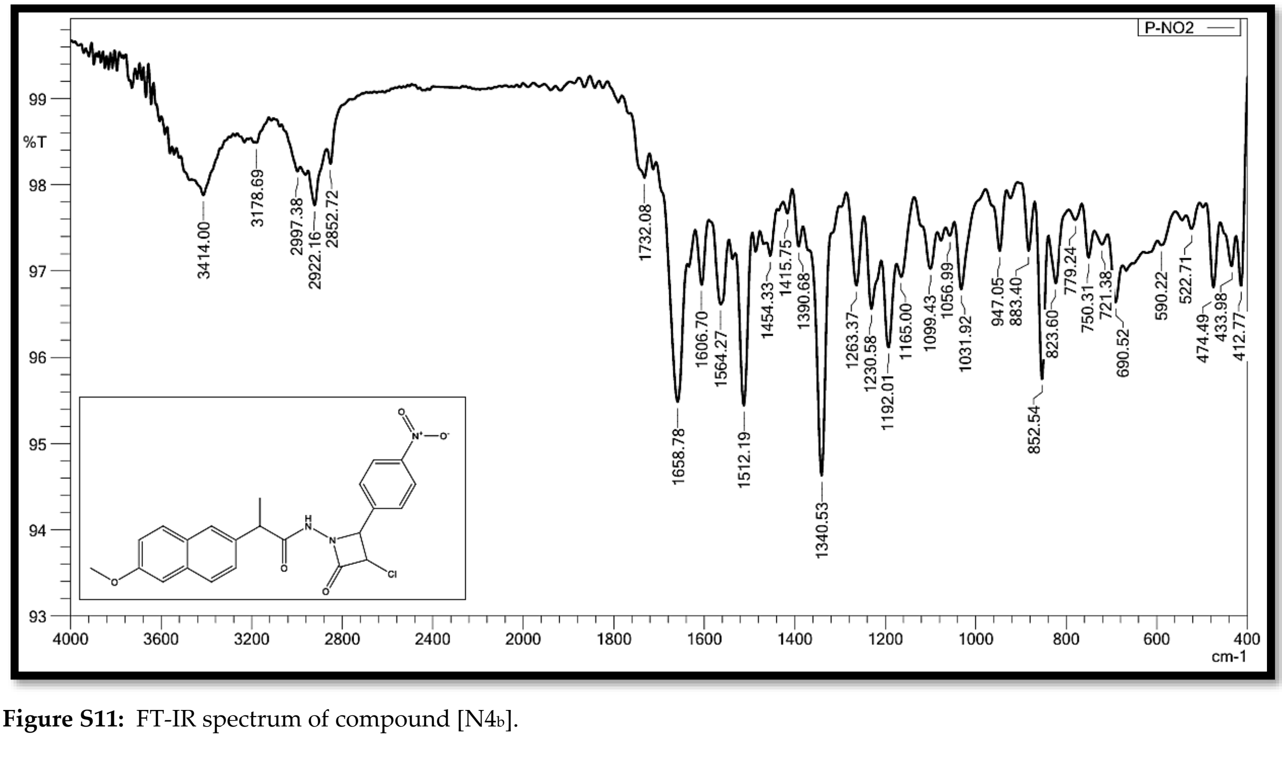

Figure S11: FT-IR spectrum of compound [N4b].

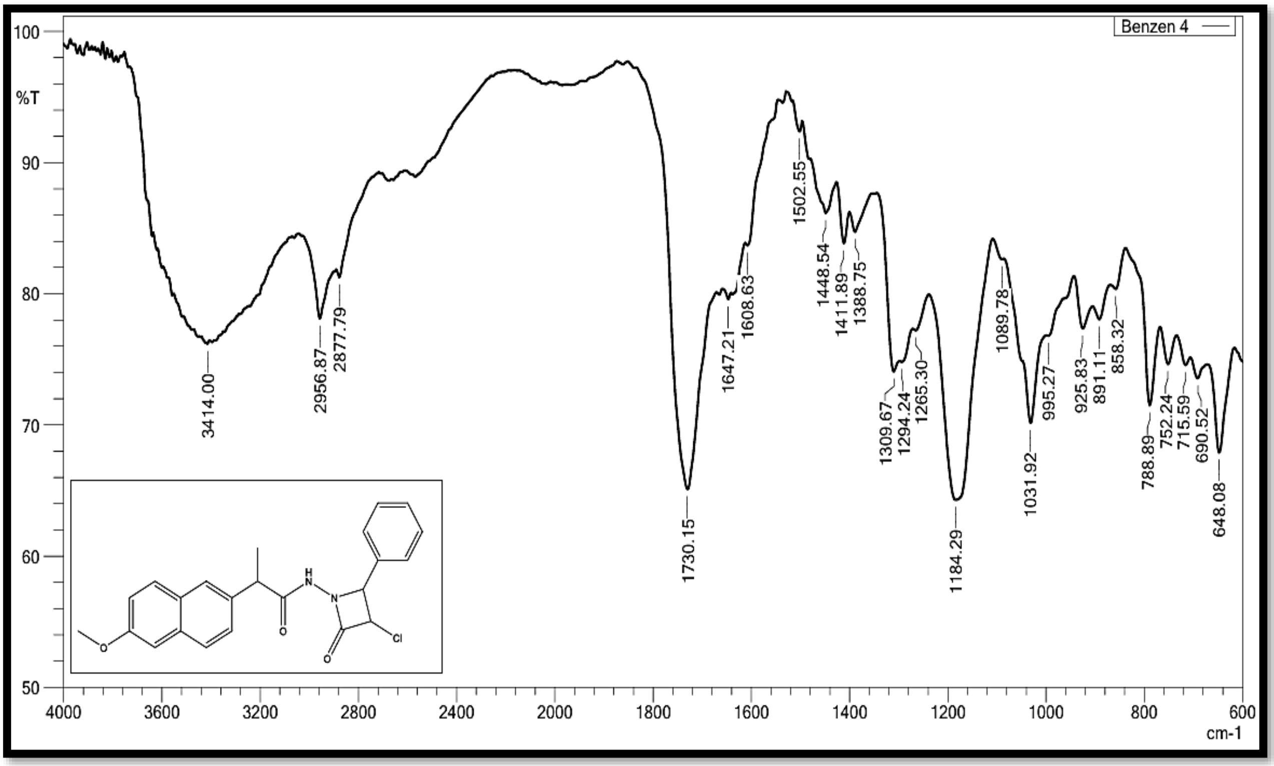

Figure S12: FT-IR spectrum of compound [N4c].

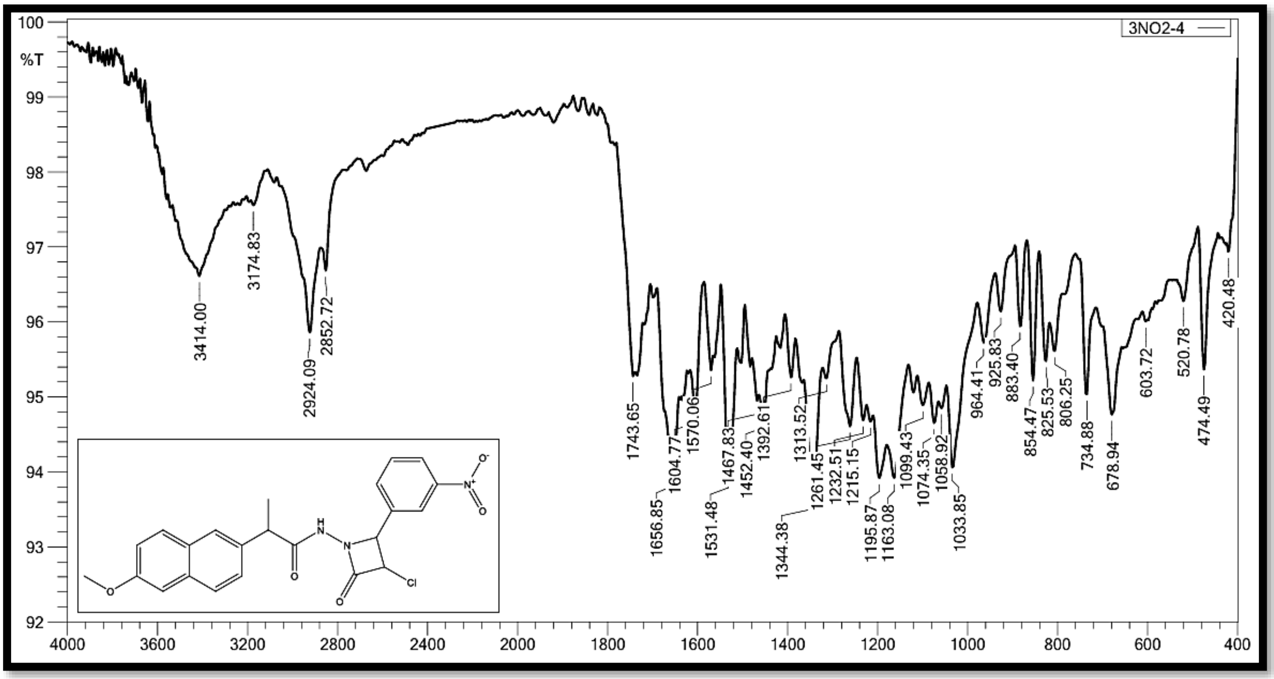

Figure S13: FT-IR spectrum of compound [N4d].

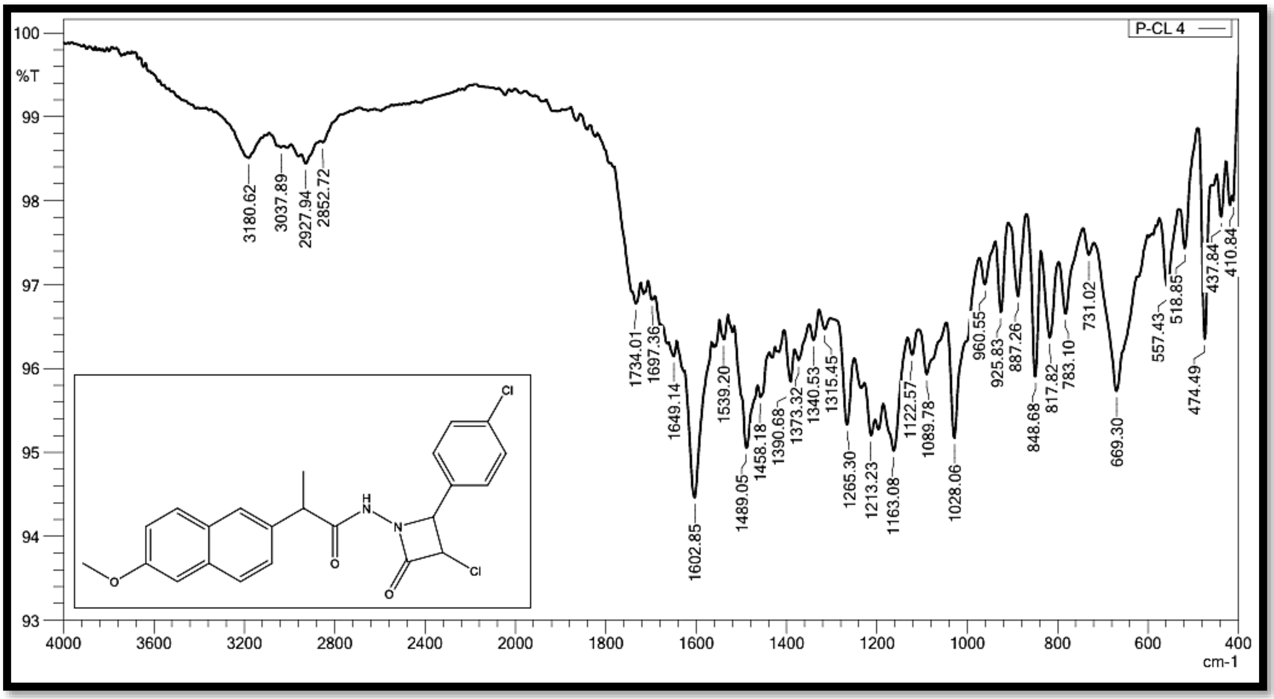

Figure S14: FT-IR spectrum of compound [N4e].

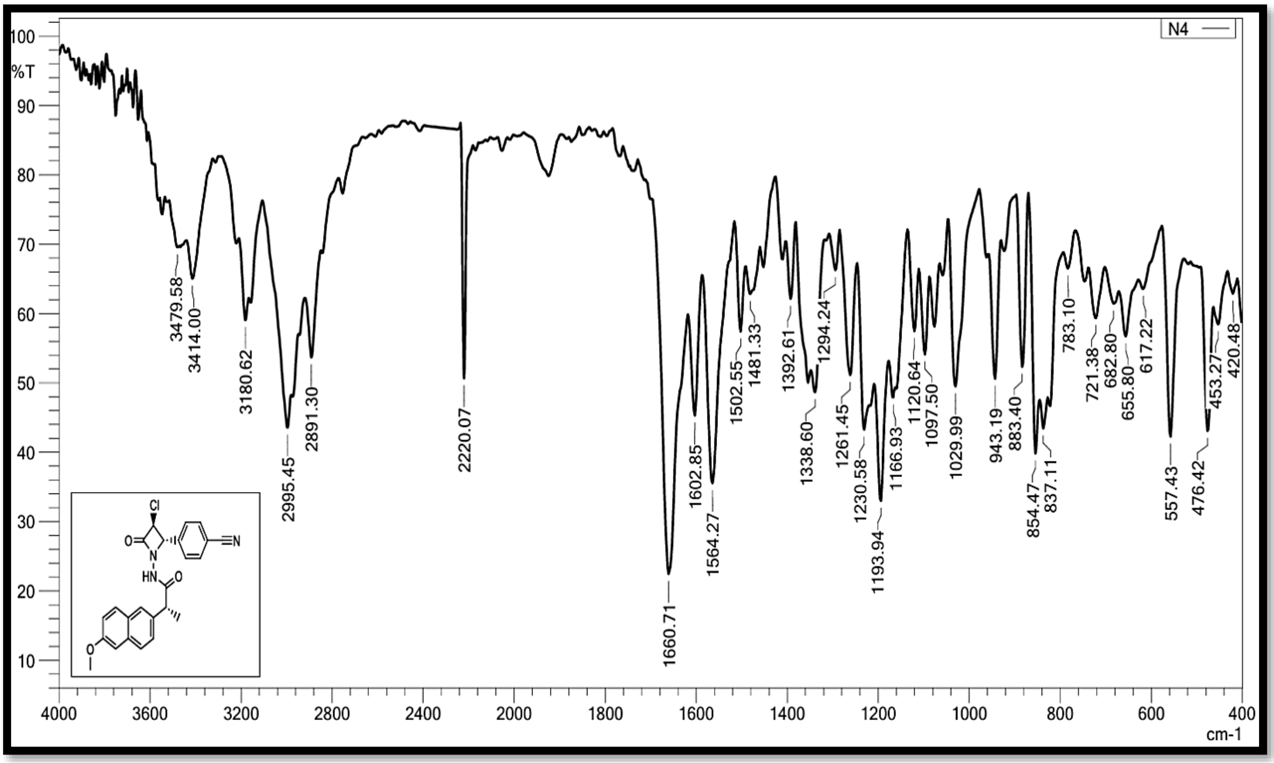

Figure S15: FT-IR spectrum of compound [N4f].

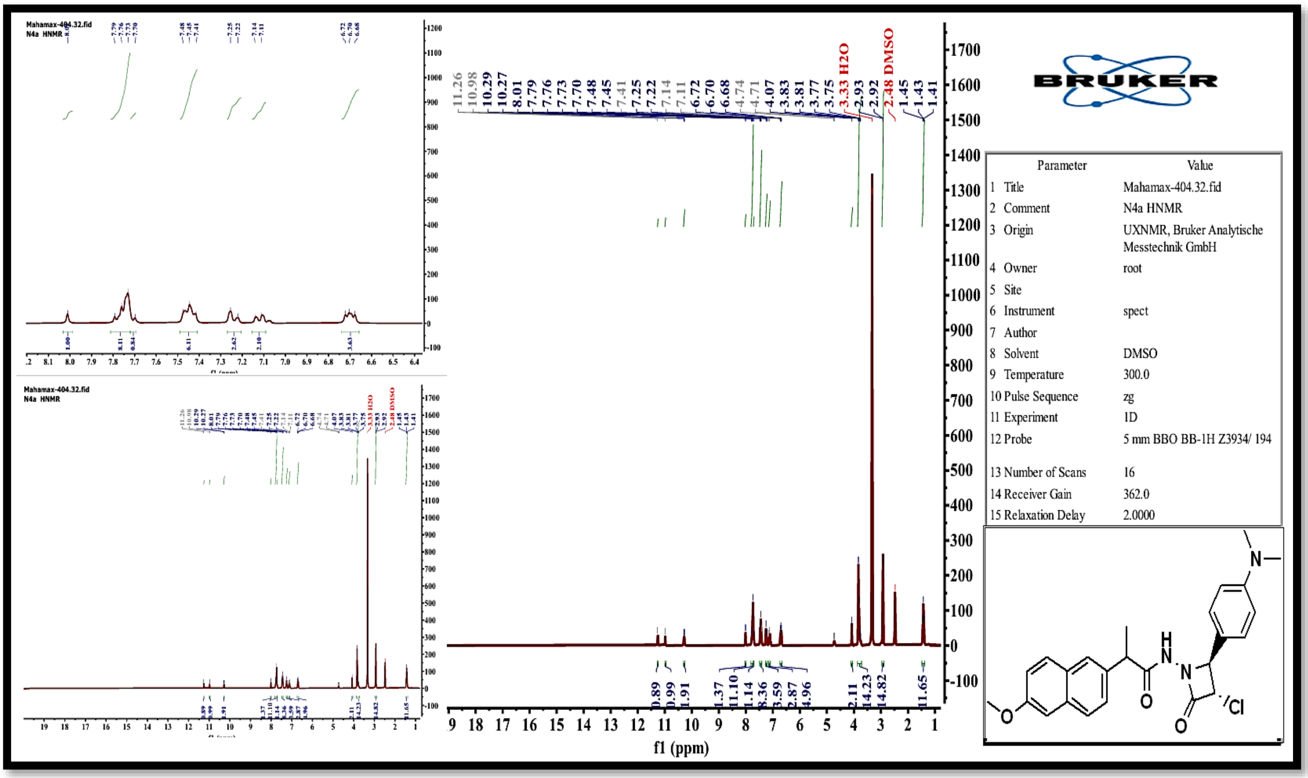

Figure S16: Compound's ^{1}H-NMR spectra (N4a)

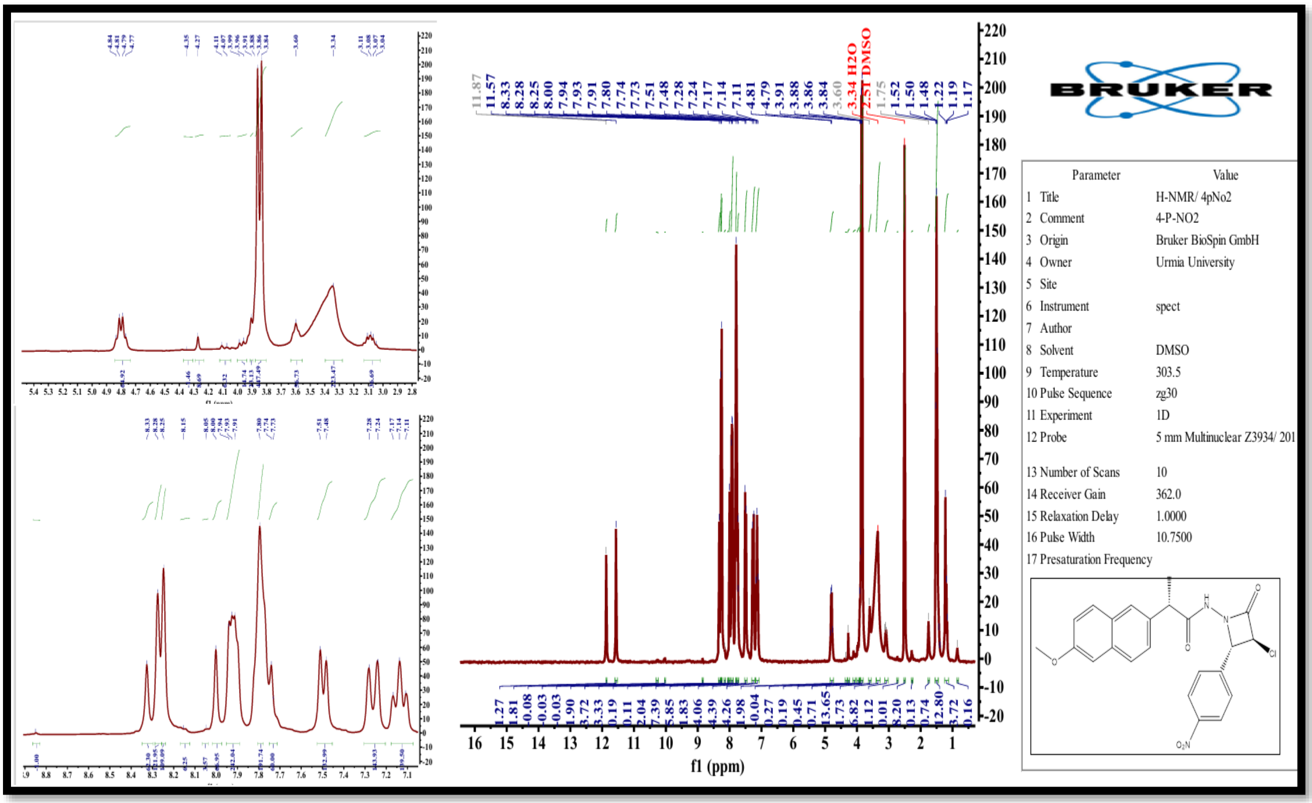

Figure S17: Compound's ^{1}H-NMR spectra (N4b)

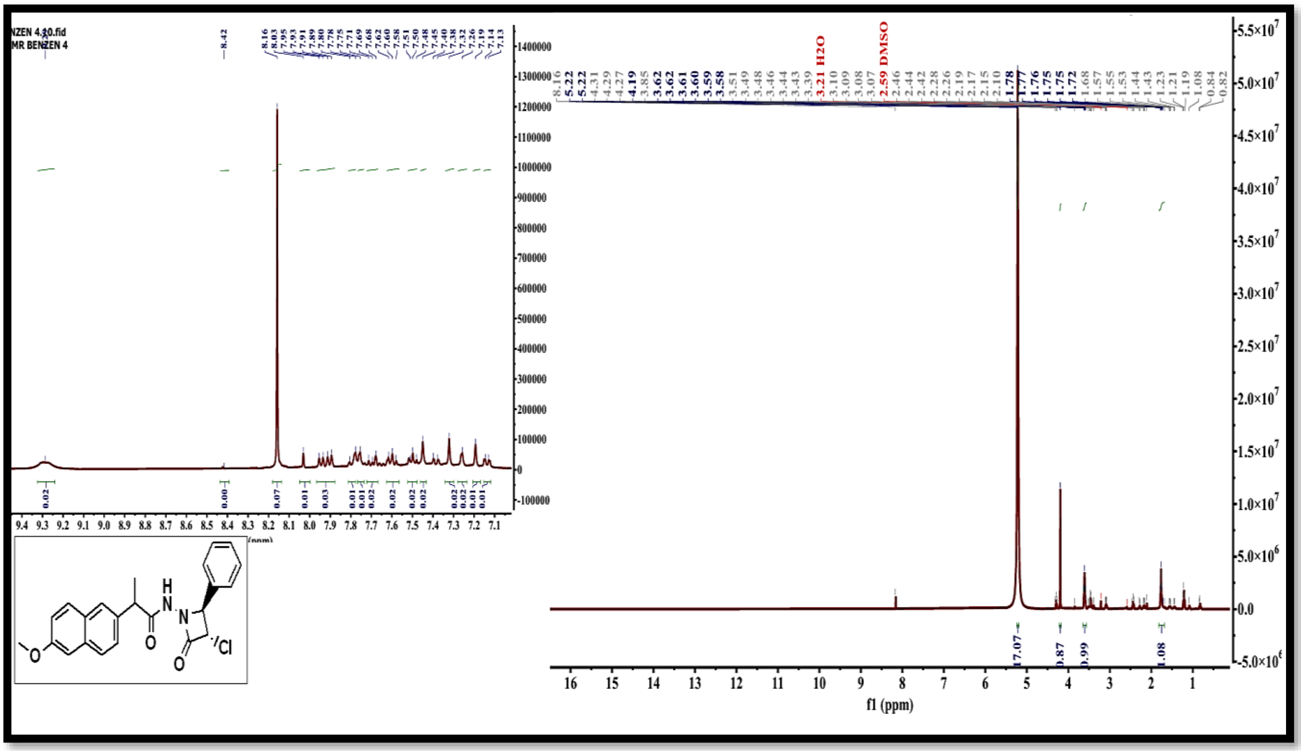

Figure S18: Compound's ^{1}H-NMR spectra (N4c)

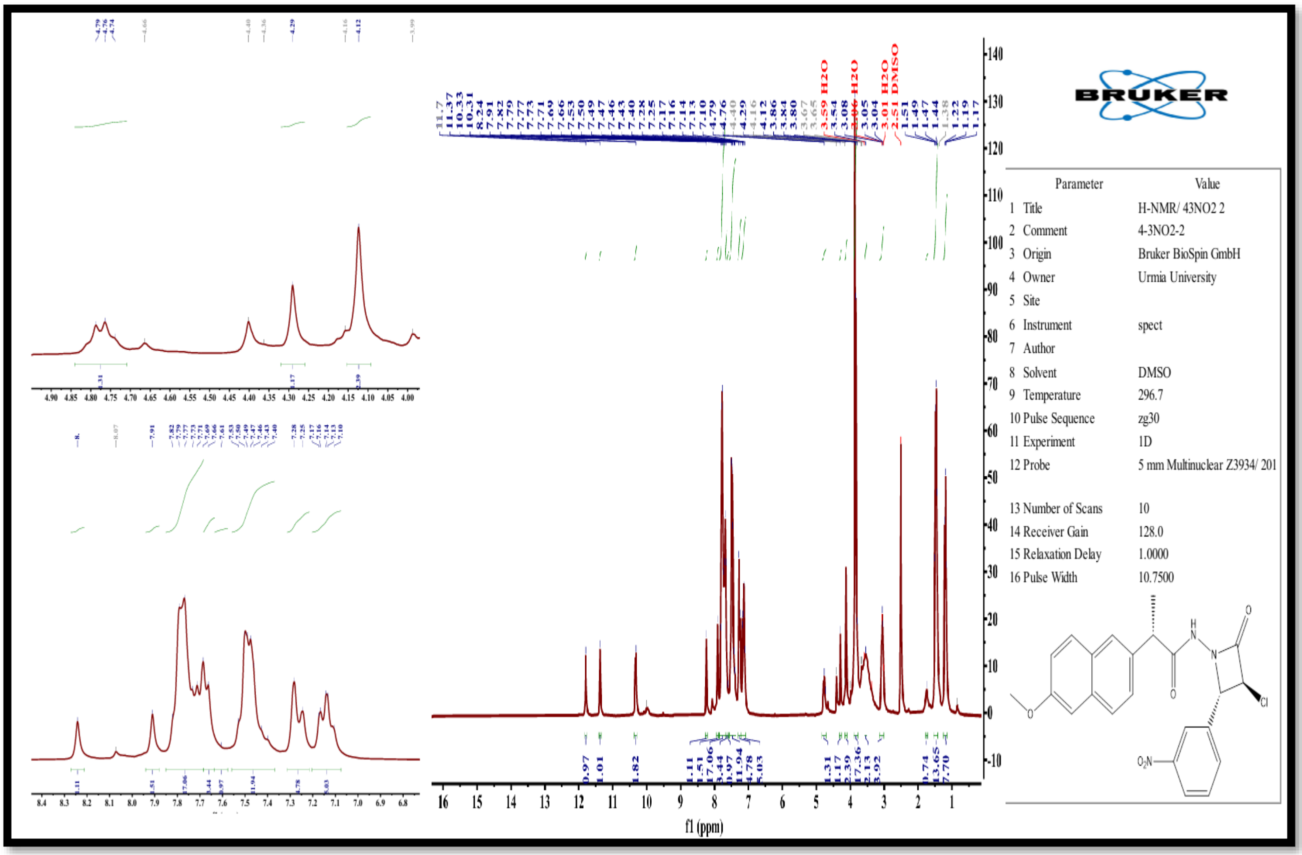

Figure S19: Compound's ^{1}H-NMR spectra (N4d

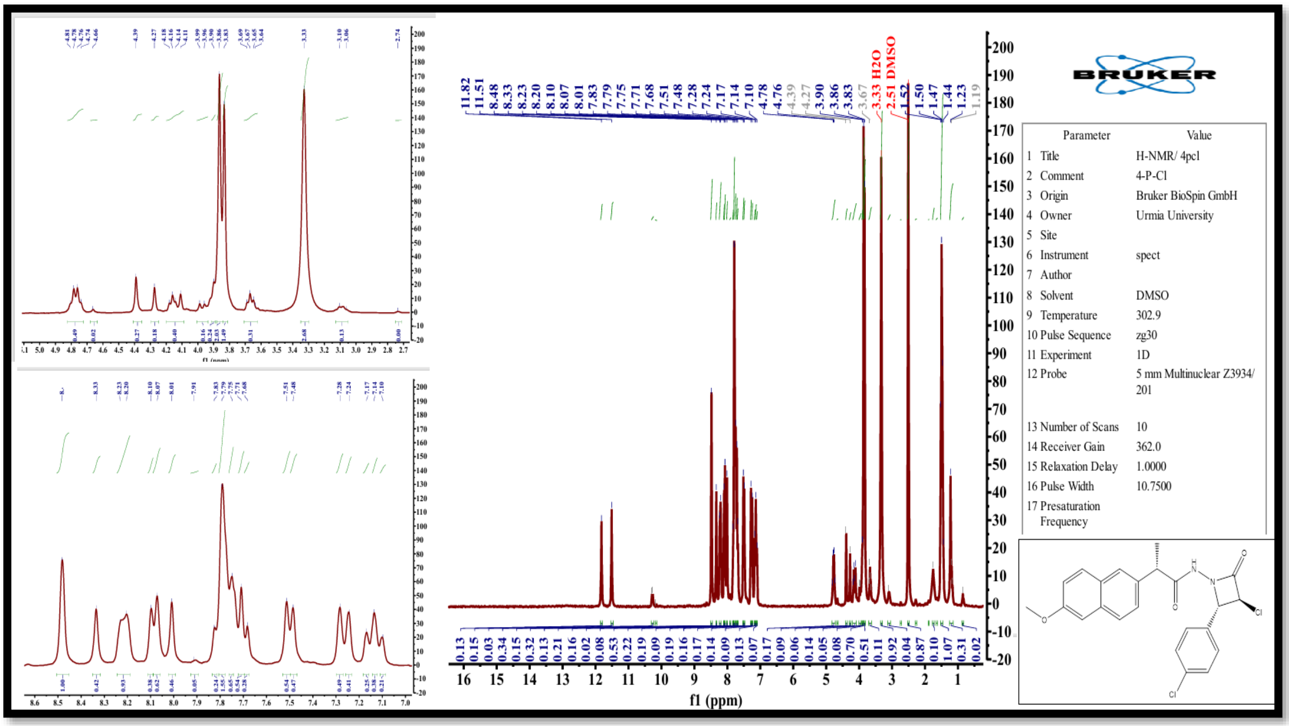

Figure S20: Compound's ^{1}H-NMR spectra (N4$_e$)

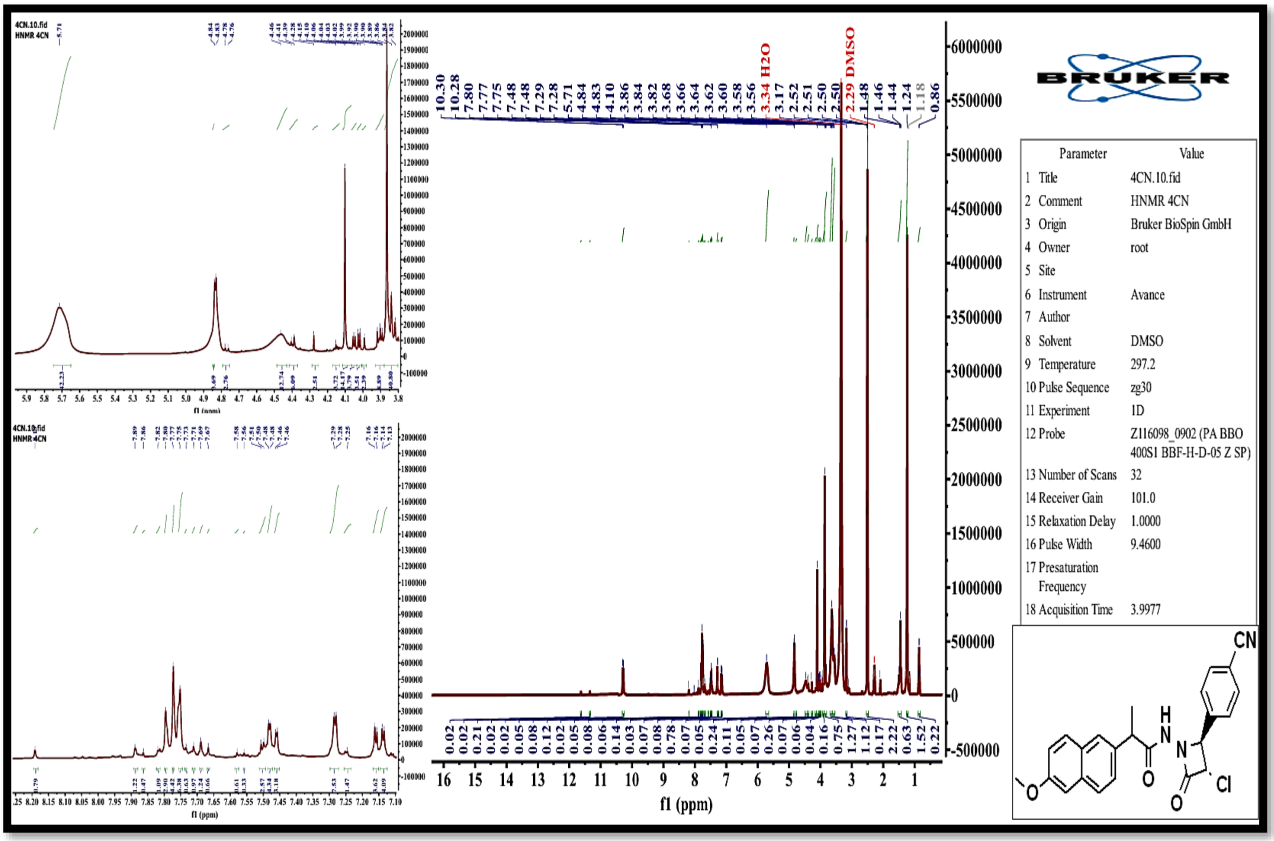

Figure S21: Compound's ^{1}H-NMR spectra (N4$_f$)

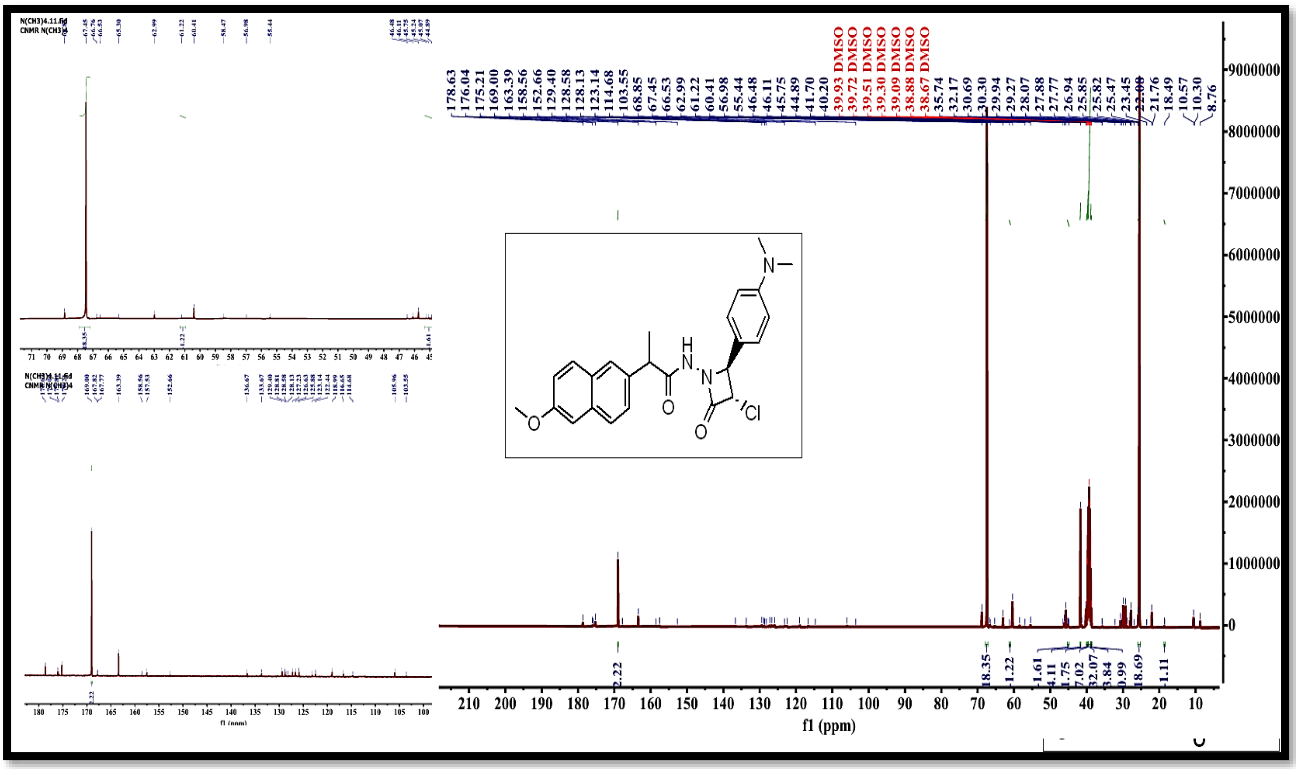

Figure S22: Illustrates the ^{13}C-NMR spectral profile of N4a.

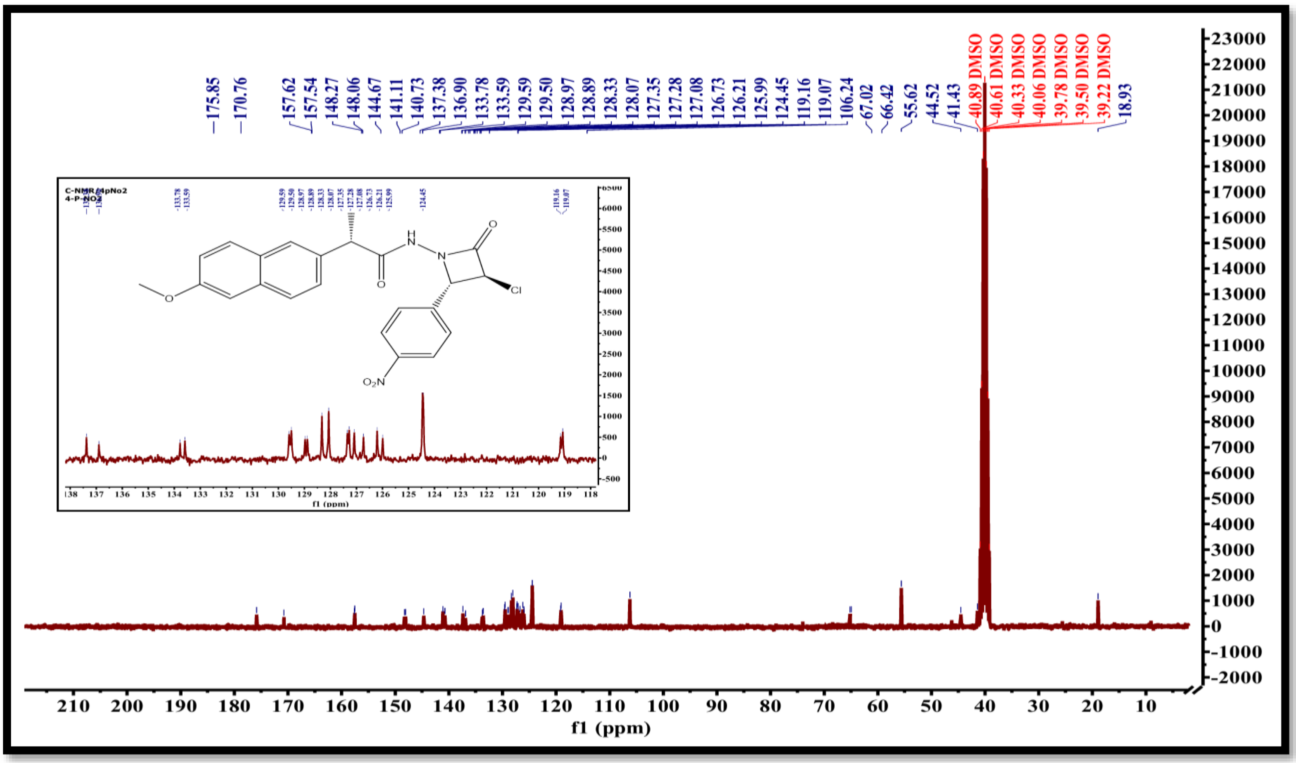

Figure S23: Illustrates the ^{13}C-NMR spectral profile of N4b

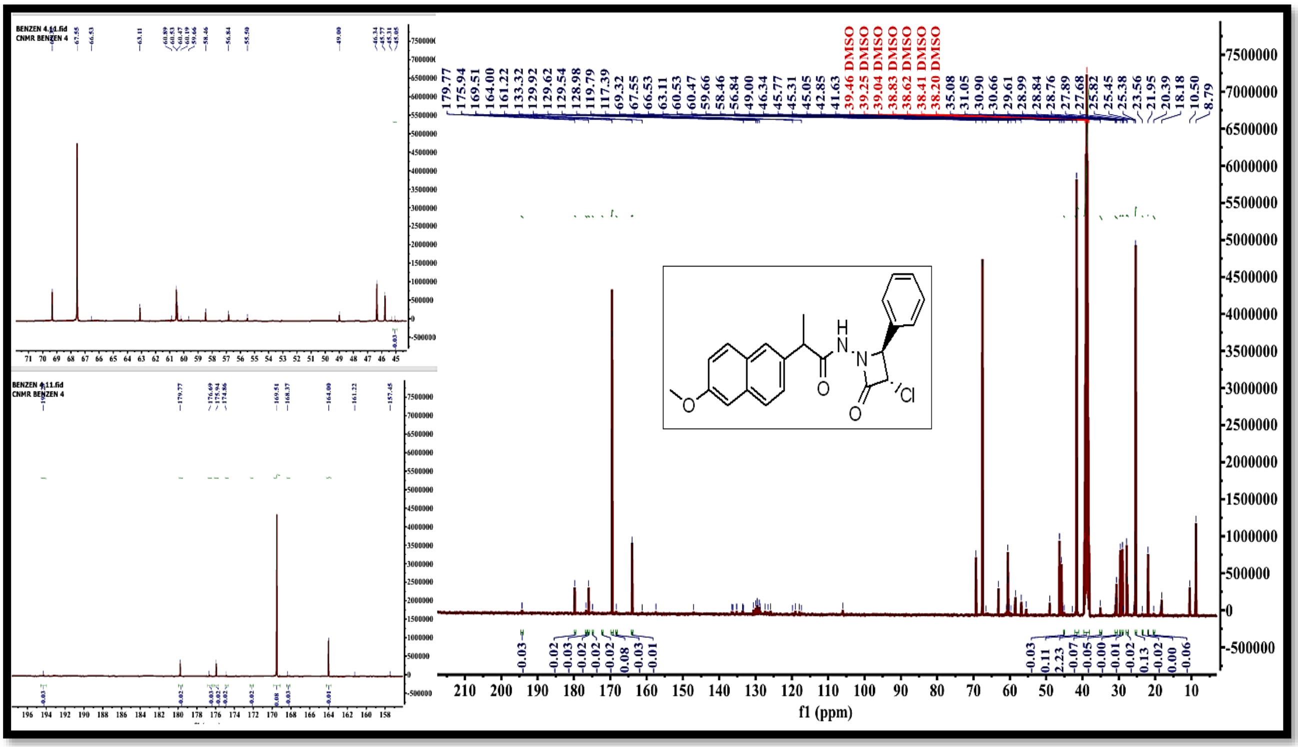

Figure S24: Illustrates the ^{13}C-NMR spectral profile of N4$_c$

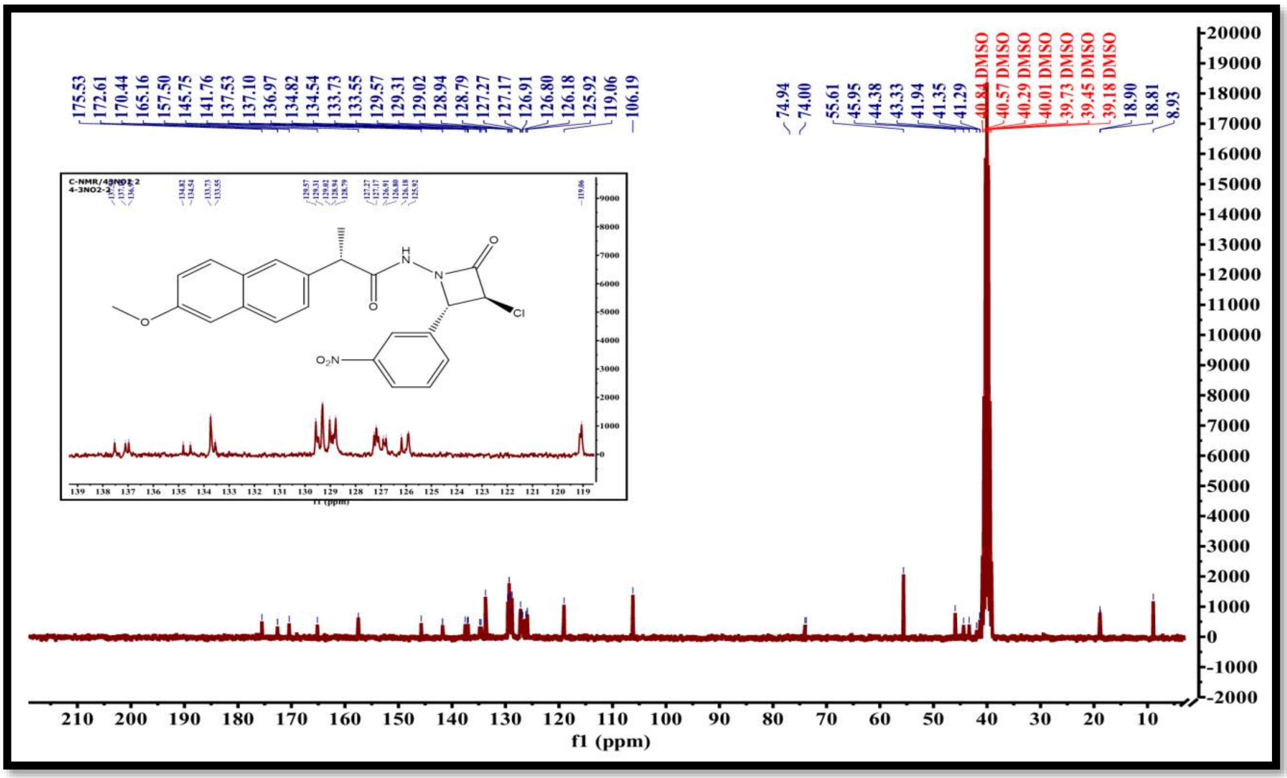

Figure S25: Illustrates the ^{13}C-NMR spectral profile of N4$_d$

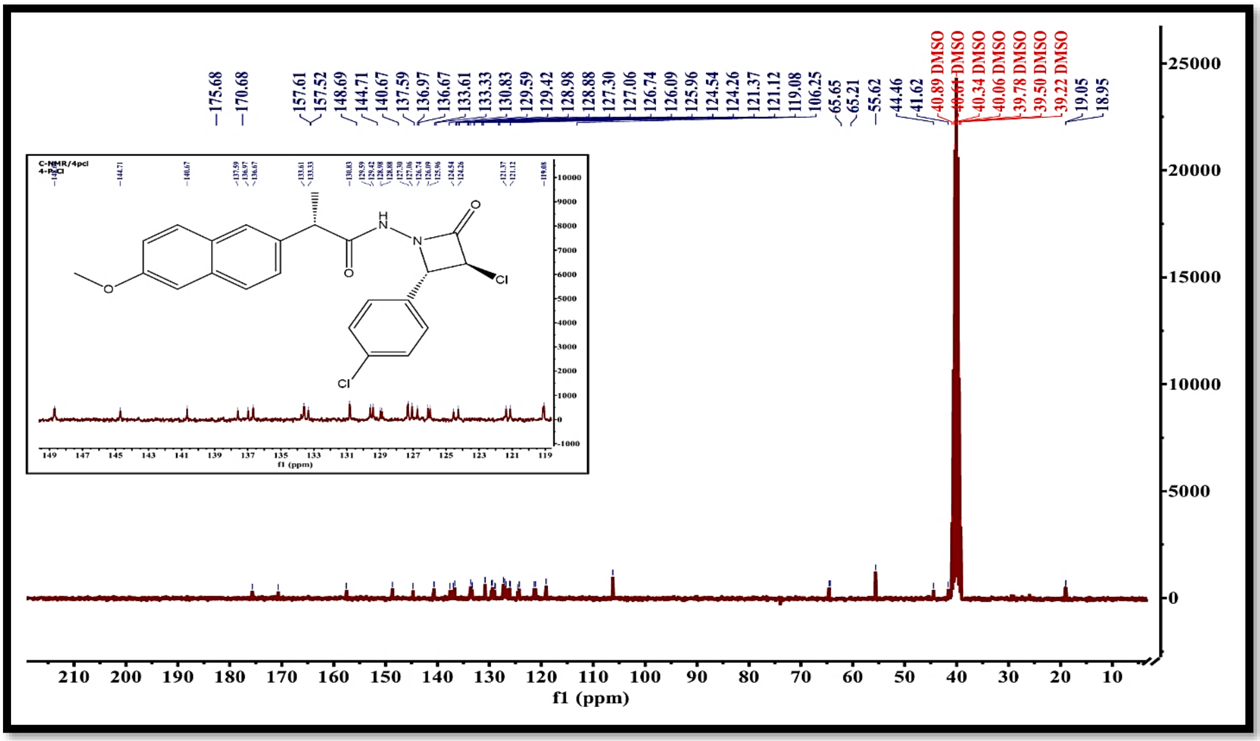

Figure S26: Illustrates the ^{13}C-NMR spectral profile of N4e

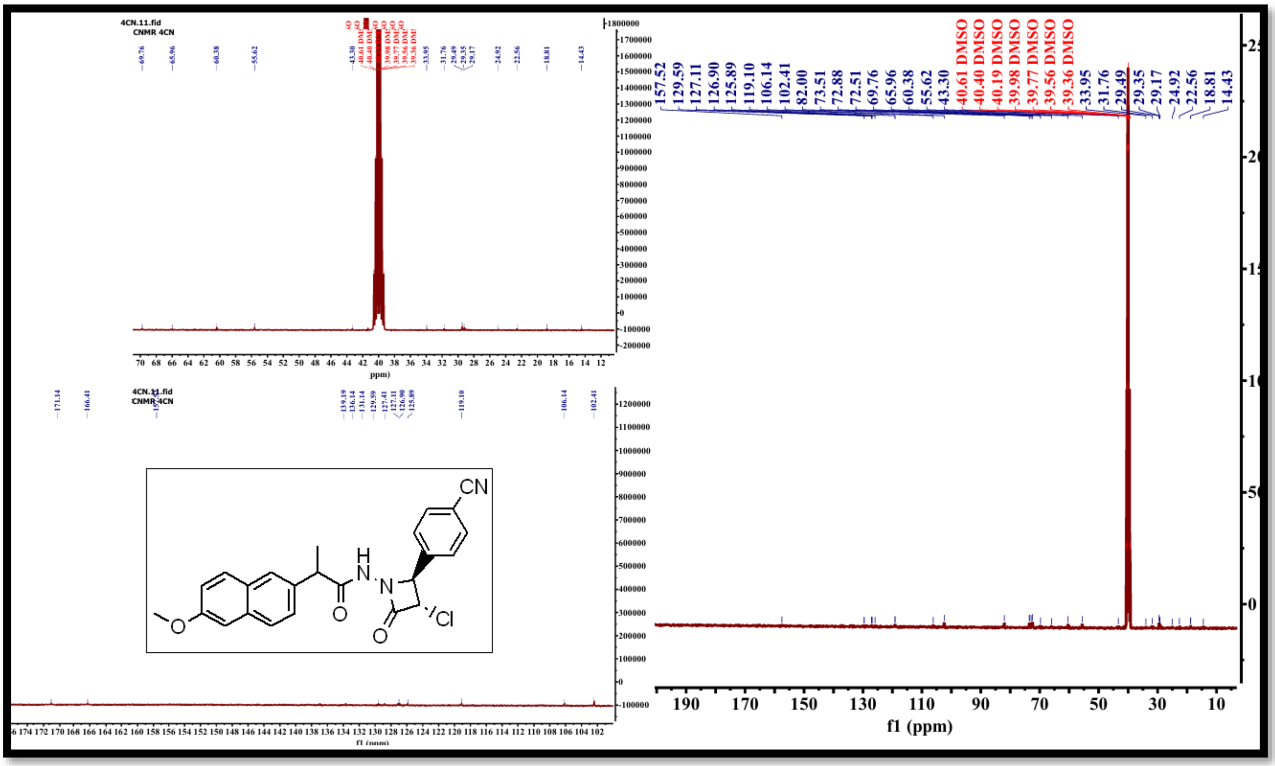

Figure S27: Illustrates the ^{13}C-NMR spectral profile of N4f
